# Indoor Environmental Quality of Residential Elderly Care Facilities in Northeast China

**DOI:** 10.3389/fpubh.2022.860976

**Published:** 2022-05-04

**Authors:** Jingyi Mu, Jian Kang

**Affiliations:** ^1^School of Architecture, Key Laboratory of Cold Region Urban and Rural Human Settlement Environment Science and Technology, Ministry of Industry and Information Technology, Harbin Institute of Technology, Harbin, China; ^2^Institute for Environmental Design and Engineering, The Bartlett, University College London, London, United Kingdom

**Keywords:** indoor environmental quality, care facility, the elderly, cold region, physical environment

## Abstract

The indoor environmental quality is based on the indoor environmental performance of buildings, such as air temperature, lighting, and acoustics. These parameters have a specific impact on users' health and experience. This study explores the relationship between the indoor environment of residential elderly care facilities in cold regions and the sensitivity of the elderly to these facilities with the aim of improving the elderly care environment. This study measured the acoustic, lighting, and thermal environment in four residential elderly care facilities in Northeast China in spring, summer, autumn, and winter through a participant survey. In the residential elderly care facilities surveyed in this study, brightness and illuminance show a nonlinear relationship with lighting evaluation. With an increase in brightness and illuminance, the satisfaction of the lighting environment in different seasons first increases and then decreases. The relative humidity of the different types of rooms varies greatly in spring and less in winter. The average air quality score of the bedroom is higher than that of the activity room. The correlation between odor assessment and overall indoor environmental quality is very poor. The results of the questionnaire survey indicate that the participants were satisfied with the facilities' overall indoor environmental quality. This quality is affected by physical, environmental, and demographic factors. This study provides a reference for the design of other residential elderly care facilities.

## Introduction

The global population is rapidly aging, which has brought unprecedented challenges such as increased medical and long-term care expenditures (1). The main function of residential elderly care facilities (RECF) is to provide living and activity, visitor, and care conditions for the elderly (2). Undoubtedly, the indoor environmental quality (IEQ) of RECF affects the physical and mental health of the elderly (3). IEQ factors mainly include the acoustic environment, indoor air quality (IAQ), temperature, lighting, visual, and auditory comfort, which impact users' comfort and health (4–7). Because the elderly spends most of their time indoors, the indoor environment has a great impact on the health of the elderly. Therefore, it is necessary to create a comfortable indoor living environment for the elderly to protect them physically and psychologically.

Excessive unwanted noise can be detrimental to health and prevent seniors in care facilities from recovering from hearing loss. Long-term exposure to an environment above 65 dB(A) can cause serious health problems such as sleep disorders, hearing loss, tinnitus, high blood pressure, and cardiovascular disease (8, 9). The elderly are more tolerant of sound but also more sensitive (10). Noise perception derives from the acoustic comfort of the surrounding environment and can reflect different evaluations of sound and soundscape (11). For many seniors, background noise and lighting interfere with sleep, cause poor concentration and physical tiredness, and interfere with normal communication. The requirements of occupants in a lighting environment change with age (12, 13). From a physiological point of view, due to the decline in retinal function, lens hardening, weakening of lens light, eye diseases, and other reasons, the elderly have higher lighting requirements (14). Additionally, because the elderly are sensitive to the surrounding temperature, it is important to maintain a constant and appropriate temperature in their living environment. When the temperature is lower than 15°C, the blood pressure of the elderly increase (15). Elderly people spend most of their time indoors. Therefore, IAQ directly affects the health of elderly people (16). Several studies have found that high CO_2_ concentration is directly related to the air pollutants concentration in indoor environment (17, 18). Sleeping in a bedroom with high levels of CO_2_ can affect sleep quality and daytime focus. When the CO_2_ concentration in the air exceeds 2,000 ppm, it causes dizziness and increases the heart rate (19). Long-term exposure to this environment can damage human health (20).

The elderly gather regularly in the activity room in care facilities. The study found that when the reverberation time in the activity hall exceeded 4 s or the sound pressure level (SPL) exceeded 65 dB (A), the subjective evaluation of the acoustic environment comfort of the elderly decreased (21). Compared with fast-rhythmed music, slow-rhythmed music has a better effect on improving the emotional state of the elderly. Under the setting of natural sounds, the pleasure brought by individual activities is significantly higher than that of collective activities (22). Indoor space layout, home facilities, and indoor environments have a significant impact on depressive symptoms in elderly individuals (23). A study of the lighting environment in special care facilities found that dynamic lighting in the living room significantly reduced the anxiety behavior of patients with dementia (24). Satisfaction with the living environment is negatively correlated with depression and positively correlated with physical activity (25). A study on the indoor thermal environment of elderly families during the Beijing heating season showed that the acceptable temperature of the rural elderly was lower than that of the urban elderly. However, acceptable temperatures may not meet the long-term health needs of the rural elderly (26).

Currently, there is a lack of research on the IAQ, acoustic environment, lighting environment, and thermal environment of RECFs in Northeast China, as well as on the relationships between these factors. A comfortable IEQ is subjective and based on individual perceptions of environmental parameters (including SPL, lighting, air temperature, relative humidity, and air quality) (27–29). It is a complex response to a building and its physical environment, which depends on the individual's physiological conditions (such as social relations, health, and financial status). Four representative indoor environment parameters were used to study the IEQ of SPL, lighting level, indoor temperature, and air quality (30–32). These parameters can be used to measure or investigate the auditory, visual, and thermal comfort and IAQ of an indoor environment.

Therefore, to improve the elderly care environment, this study focused on six issues:

- How satisfied are the elderly with the indoor environmental quality of RECF? The main items to be investigated included the acoustic environment, lighting environment, thermal environment, and IAQ.
- What is the relationship between different physical environmental factors and overall IEQ evaluation?
- How do elderly people with different demographic and social backgrounds evaluate RECF differently?
- It is expected that this research will provide theoretical support for the design of RECF indoor environments.

## Methods

In this study, the SPL, illuminance, brightness, temperature, humidity, and IAQ of the activity room, bedroom, restaurant, corridor, and consultation room of four RECFs were measured. A survey was administered to collect evaluations of the elderly of the RECFs' physical environment factors. The experimental process is illustrated in Supplementary Figure 1.

### Sites

We conducted field surveys in four RECFs in Harbin, Changchun, and Shenyang, the capital cities of China's three northeastern provinces, from spring to winter 2018. Harbin, Shenyang, and Changchun have mid-temperate continental monsoon climates. The annual average temperature in Harbin is 4.5°C, the average temperature in January in winter is approximately −19°C, and the average temperature in July in summer is approximately 23°C. The annual average temperature in Shenyang is 8°C, the average temperature in January in winter is approximately −11°C, and the average temperature in July in summer is approximately 25°C. The average temperature in Changchun is 5.5 °C, the average temperature in January in winter is approximately −15°C, and the average temperature in July in summer is approximately 24°C. The details of these four RECFs are shown in Table 1. The floor plans and photographs are shown in Supplementary Figures 2, 3, respectively.

**Table 1:** Profiles of the four facilities.

	**HGD**	**GX**	**SHQ**	**AD**
City	Harbin	Changchun	Shenyang	Shenyang
Season	Spring	Spring	Spring	Spring
	Summer	Summer	Summer	Summer
	Autumn	Autumn	Autumn	Autumn
	Winter	Winter	Winter	Winter
Number of beds	50	200	180	350
Number of activity rooms	2	10	4	3
Street facing	√			
Reconstruction	√	√		√
Location	City center	City center	Suburb	New district
Building structure	Brick and Concrete Structure			
Heating	Centralized heating			
Window structure	Double layer sealed tempered glass			
Ventilation	Natural ventilation			
Window to wall ratio	0.32	0.41	0.26	0.36
Heat transfer coefficient	2.0	1.9	2.1	2.0
Shape coefficient	0.30	0.34	0.30	0.31

Although RECFs differ in many important aspects, all establishments provide accommodations, meals, laundry, activity rooms, and medical services for the elderly. Subsequently, probability (stratified) sampling was used to select samples (33). Small RECFs were defined as facilities with < 150 beds, medium RECFs as facilities with 151–300 beds, large RECFs as facilities with 301–500 beds, and super-large RECFs as facilities with over 500 beds. These divisions are defined in the building design codes for senior facilities in China.

We named the four RECFs investigated in this study HGD, GX, SHQ, and AD, which are present in Harbin, Changchun, and Shenyang, respectively. Details are presented in Table 1. The research and survey seasons were spring, summer, autumn, and winter of 2018. The spring test was conducted from 15 April to 20 May; summer test was conducted from 20 July to 15 August; autumn test from 13 October to 9 November; winter test from 5 January to 15, and 28 January to 8 February (for more information, see Table 1).

### Participants

The elderly at the facilities were asked to participate in a survey, and information on their backgrounds, such as age, gender, and education, and their satisfaction with the IEQ indicators of the RECFs were collected. The final analysis only included surveys completed by residents who had lived in these RECFs for 6 months or more. According to Rockwood et al.'s frailty scales, the elderly who scored between 1 and 4 qualified as participants in this survey, meaning that their physiological and psychological status was sufficient to participate in the study (34). According to Rockwood et al.'s frailty scale, a score of 1 represents very fit, 2 represents well, 3 represents well with treated comorbid disease, and 4 represents apparent vulnerability. Overall, the elderly with scores between 1 and 4 had a better physical condition. According to the frailty scale, the elderly selected in this study were all healthy and autonomous. As noted above, 885 surveys were collected, 315 in spring, 94 in summer, 350 in autumn, and 126 in winter. Of these, 408 (46.1%) were men and 477 (53.9%) were women (Supplementary Figure 4).

### Measured Indoor Environment

In terms of the types of rooms tested and the parameters studied, eight bedrooms, six activity rooms, four restaurants, four corridors, and four consultation rooms were sampled in the four RECFs, with sample measurements including eight major indoor environmental parameters: SPL, brightness and illuminance levels, temperature, relative humidity, CO, CO_2_, and O_2_. The physical environment test consisted of two parts: a continuous test in a fixed room, and an instant test at the end of the questionnaire.

**Figure 1:**
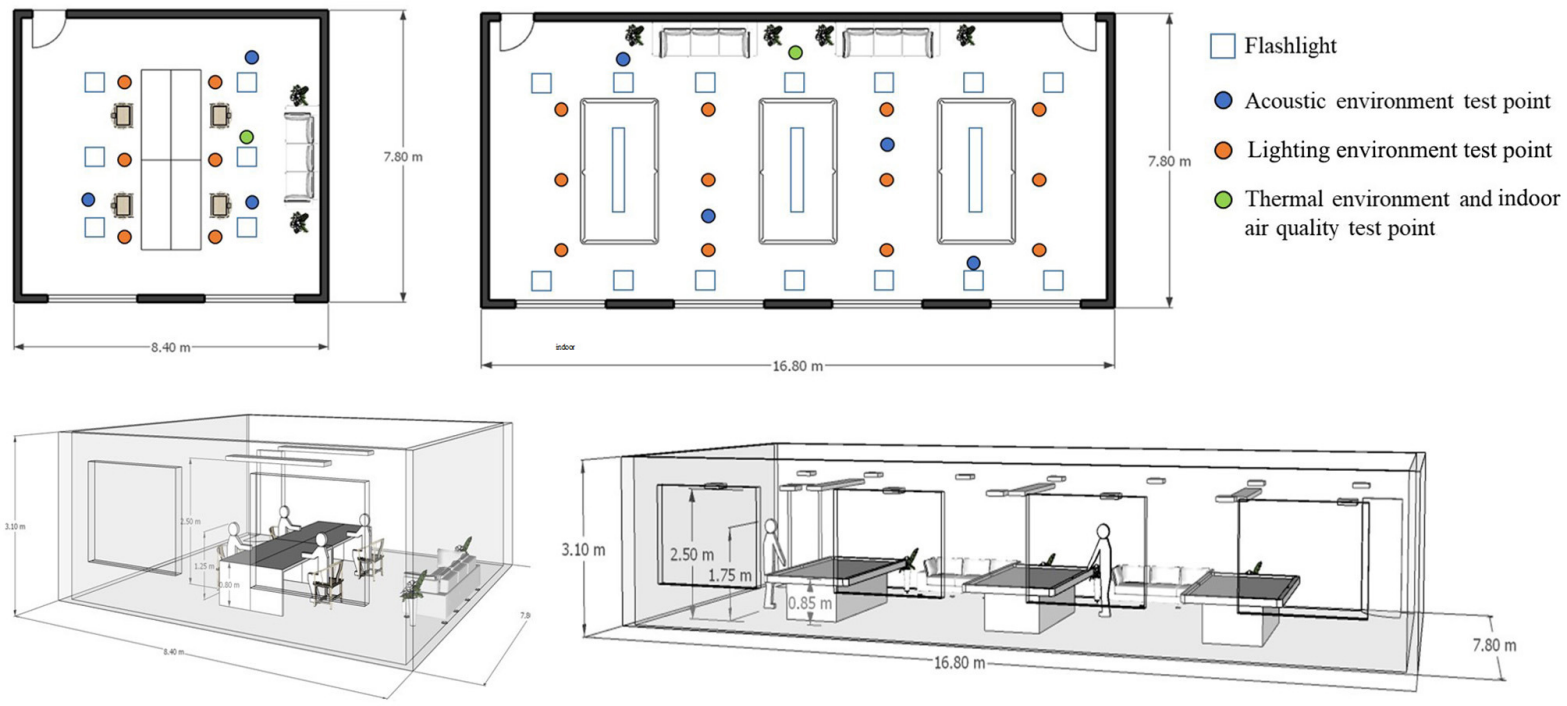
Layout of the test points.

A multipoint layout method was adopted for each room. Six rows of measurement points were arranged along the long axis and three columns of measurement points were arranged along the short axis in each room. Each replicate was 1.2 m from the floor, and at least 1.0 m from the walls and windows. The physical environments of the four RECFs were tested and a survey was conducted during each season. The test time for each room was from 8:00 to 22:00 at intervals of 1 h. The test contents, instruments, test ranges, and accuracies are listed in Table 2. For the thermal environment, a Center 314 temperature/humidity data logger was used to measure the air temperature and relative humidity. Taking illuminance (lx) and luminance (cd/m^2^) as parameters for evaluating the luminous environment, T-10A and GPH-1001 lighting environment measuring instruments were used to collect 20 sets of data continuously, and then 20 sets of data at 1 h intervals until completion. The standing test height was 1.6 m, and the sitting test height was 0.9 m. The detailed test process is illustrated in Figure 1. SPL was used as the parameter to evaluate the acoustic environment, and it was recorded using an 801-sound level meter. In each season, each room was tested once, and each test lasted for 2 days. The background noise of the fixed room was between 30–35 dB(A). Finally, hourly data were averaged and plotted. During the measurements, the sound level meters were set at low speed. The distance from the measurement site to the wall and other reflective surfaces was at least 1 m. The distance from the measurement site to the ground was between 1.2 m and 1.5 m. Illuminance and brightness were measured at the height of the line of sight of the survey participants. The environmental variables at the sites were continuously recorded for 20 min, after which average values were calculated. The thermal environments of temperature and humidity were measured following ISO 7726. The indoor CO, CO_2_, and O_2_ concentrations were measured using calibrated fast-response digital instruments (MS500-5). According to the recommendation of ISO 7726, data were recorded every 5 min at an altitude of 1.1 m. The four environmental parameters were measured during the same period and on the same date. The acoustic and lighting environments were measured using hand-held instruments, and the thermal environment and IAQ were measured by placing the instruments in fixed positions. Supplementary Figure 5 shows a photograph of the test process.

**Table 2:** Measurements and instruments used in the study.

**Test**	**Instrument**	**Test Range/Accuracy**
Acoustic	801 Sound Level Meters	19 dB(A)−137 dB(A) [±0.1 dB(A)]
Illuminance	T-10A Illuminance Meters	0.01–299,000 lx (±5%)
Brightness	GPH-1001 luminance Meters	20 cd/m^2^-2000 kcd/m^2^
Temperature	K-type thermocouple (Center 314 Temperature/Humidity Datalogger, Center Tech, Taipei, Taiwan)	−40–80°C (±0.1°C);
Relative humidity (RH)	RH sensor (Center 314 Datalogger)	0–99% (±3%)
CO		0–1000 ppm (± 0.1 ppm)
CO_2_	Composite gas detector MS500-5	0–5000 ppm (± 1 ppm)
O_2_		

After the questionnaire survey, the IEQ of the rooms investigated was tested. Ten repeated tests were performed in the lighting environment and the average of the results was taken. The acoustic environment was tested three times using an 801-sound level meter, and 10 sets of readings were automatically recorded each time. The SPL readings were analyzed using the A-weighted mean value. If a sudden noise occurred during the test, the IEQ test was repeated. The thermal environment read the corresponding test values in the instrument according to location and time, and the results were averaged. IAQ was analyzed using portable instruments.

### Survey

In this study, a survey was used to explore the subjective perception and satisfaction of occupants of RECFs on the building's acoustics, lighting, thermal comfort, IAQ, and overall IEQ. The survey was compiled with reference to surveys in studies with similar goals and designs (35, 36) and then tested and modified through pilot surveys. The survey was piloted with 30 elderly individuals from a RECF in Harbin, independent of those included in this study. In the pilot survey process, a face-to-face survey was conducted to improve the return rate. Information was divided into two main categories: background information and satisfaction with IEQ (see Supplementary Table 1). Each participant completed the survey to 1–3 times per day.

### Statistical Analysis

SPSS 20.0 was used for statistical analysis. Pearson correlation analysis and linear regression analysis were used to determine the relationship between the elderly's evaluation of indoor environmental comfort and the four environmental factors, and the mean difference was used to investigate the influence of seasons and regions on RECF indoor environmental changes. *Statistical significance was set at p* < *0.05*.

## Results and Discussion

### Evaluation of the Acoustic Environment

#### Sound Pressure Level

**Figure 2:**
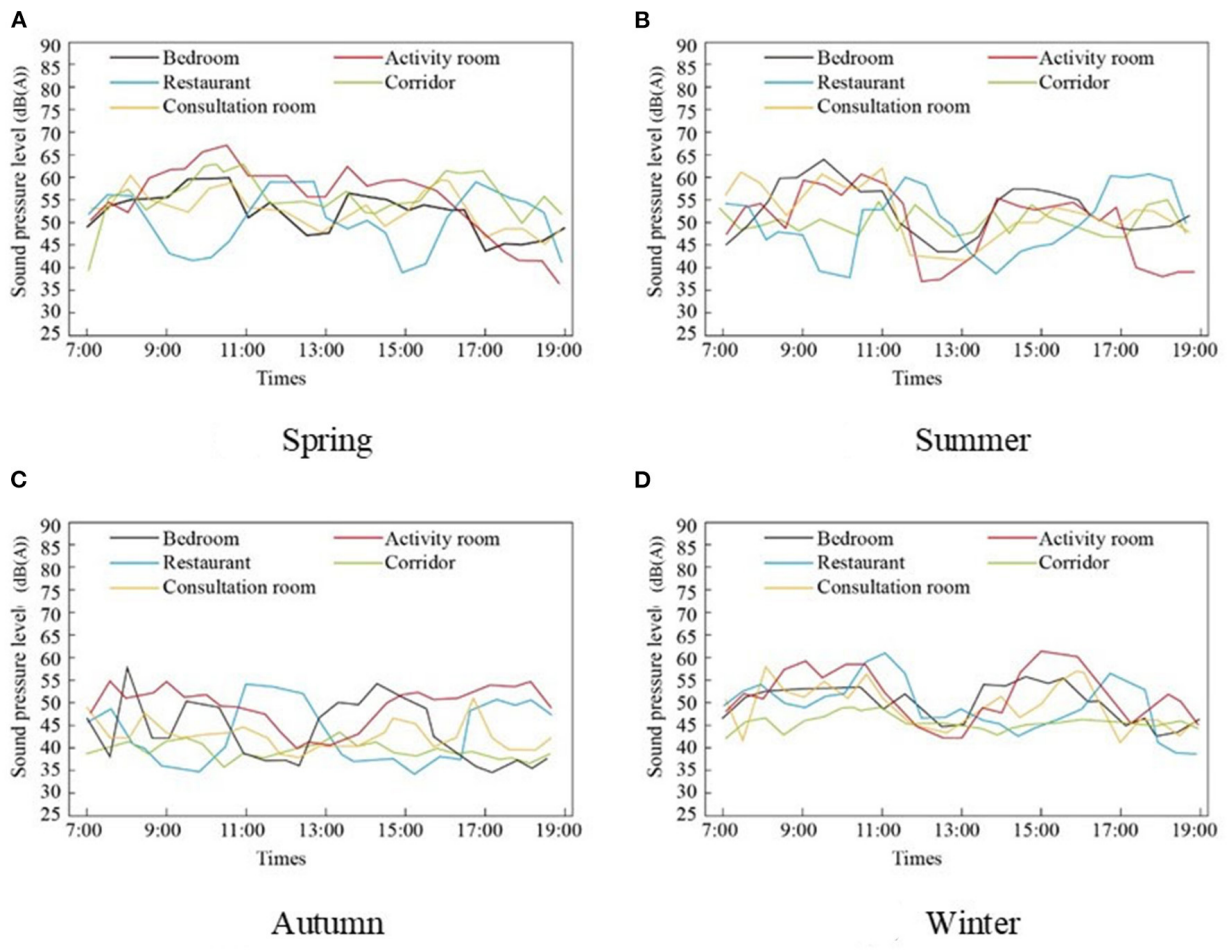
SPL measurement in different seasons and different rooms. **(A)** Spring, **(B)** Summer, **(C)** Autumn, and **(D)** Winter.

As shown in Figure 2, the overall SPL in spring was higher than the overall SPL in autumn. The SPL of the activity room exceeded 65 dB(A) in spring. The SPL of the restaurant shows a regular change, which is higher between 7:00 and 8:00, 11:00 and 13:00, and 17:00 and 18:00. This finding is consistent with the dining habits of the elderly. The SPL of the activity room fluctuated the most in summer, with relatively small changes in spring, summer, and winter, and showed the opposite trend to that of the restaurant.

According to the Chinese industry standard “Architectural Design Standards for Elderly Care Facilities JGJ450-2018,” where the occupants are elderly, have excellent sound insulation and noise prevention devices, the noise in the living environment should be <40 dB(A), the air sound insulation should not be <50 dB(A), and the impact sound should not exceed 75 dB(A) (37). The noise levels of the RECFs in this study were slightly higher than stipulated, with a median level between 50 dB(A) and 60 dB(A). This difference may depend on specific sound sources in each RECF, the different behaviors of employees and the elderly, and traffic noise (38). Additionally, some employees occasionally walking in corridors can generate noise (for example, night shifts or caring for the elderly) (38). From this perspective, the surveyed RECFs need to strengthen noise control.

#### Evaluation of Acoustic Factors

**Figure 3:**
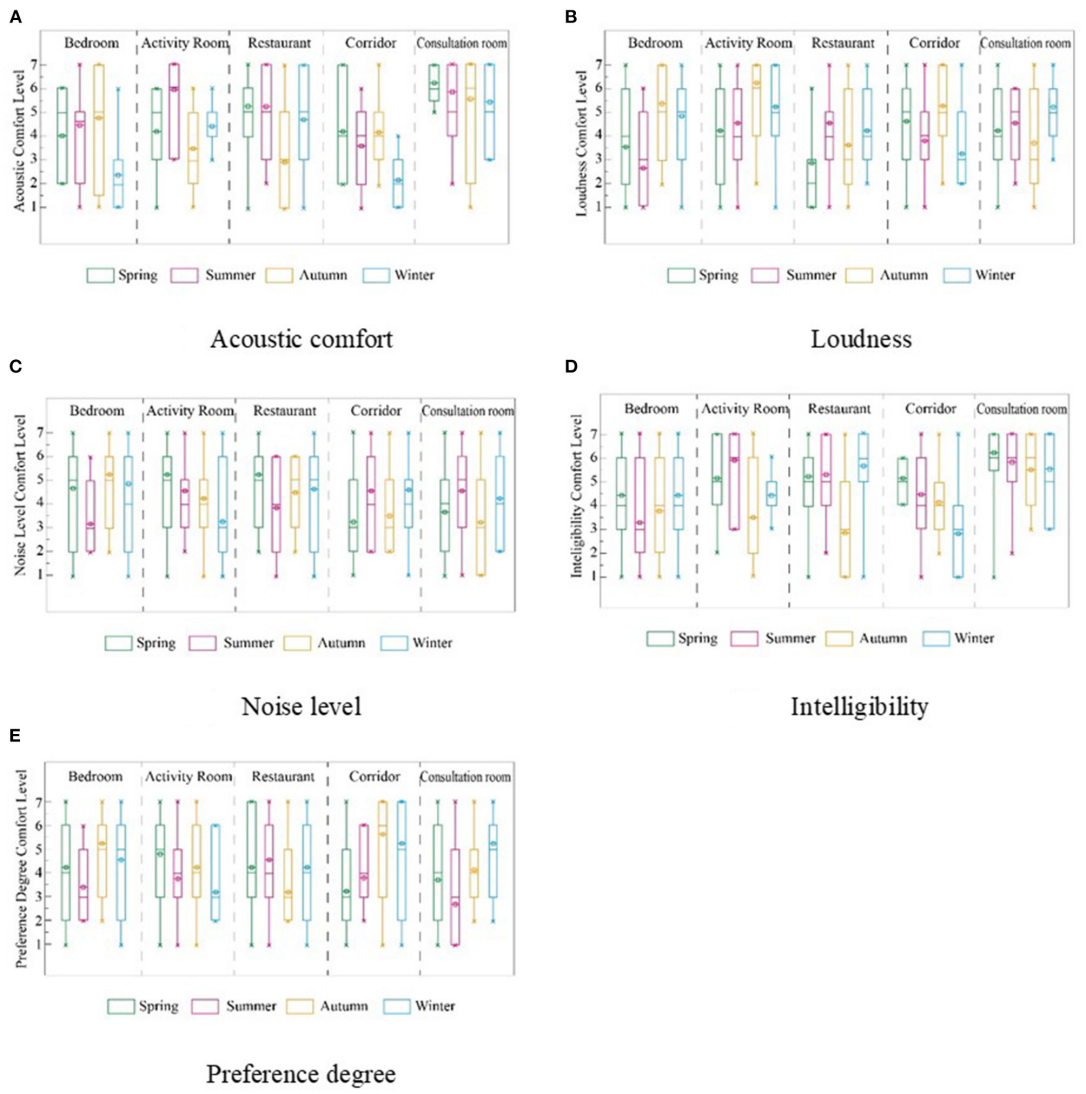
Evaluation of acoustic indicators of 4 RECFs based on seasonal differences. **(A)** Acoustic comfort, **(B)** Loudness, **(C)** Noise level, **(D)** Intelligibility, and **(E)** Preference degree.

According to the evaluation results (Figure 3), the acoustic comfort level scores of bedrooms, activity rooms, restaurants, and corridors are almost all lower than 5 points in the four seasons, indicating that the acoustic environment of these types of rooms is poor.

Generally, the acoustic environment of the four RECFs was evaluated at a medium level (3.5–5 points). According to the seasonal change survey, there was no obvious change trend for acoustic indicators, nor were the evaluation results in bedrooms and activity rooms affected by seasonal changes. Autumn's assessment value was more than 1% lower than those in summer and winter. This was because when the survey was conducted in autumn, the surrounding buildings were under construction, and the resulting noise affected the evaluation of the acoustic environment. The overall acoustic environment change was not significantly affected by room type and season, but the overall score was not high; therefore, the acoustic environment of RECF should be strengthened.

#### Relationship Between SPL and Acoustic Evaluation

**Figure 4:**
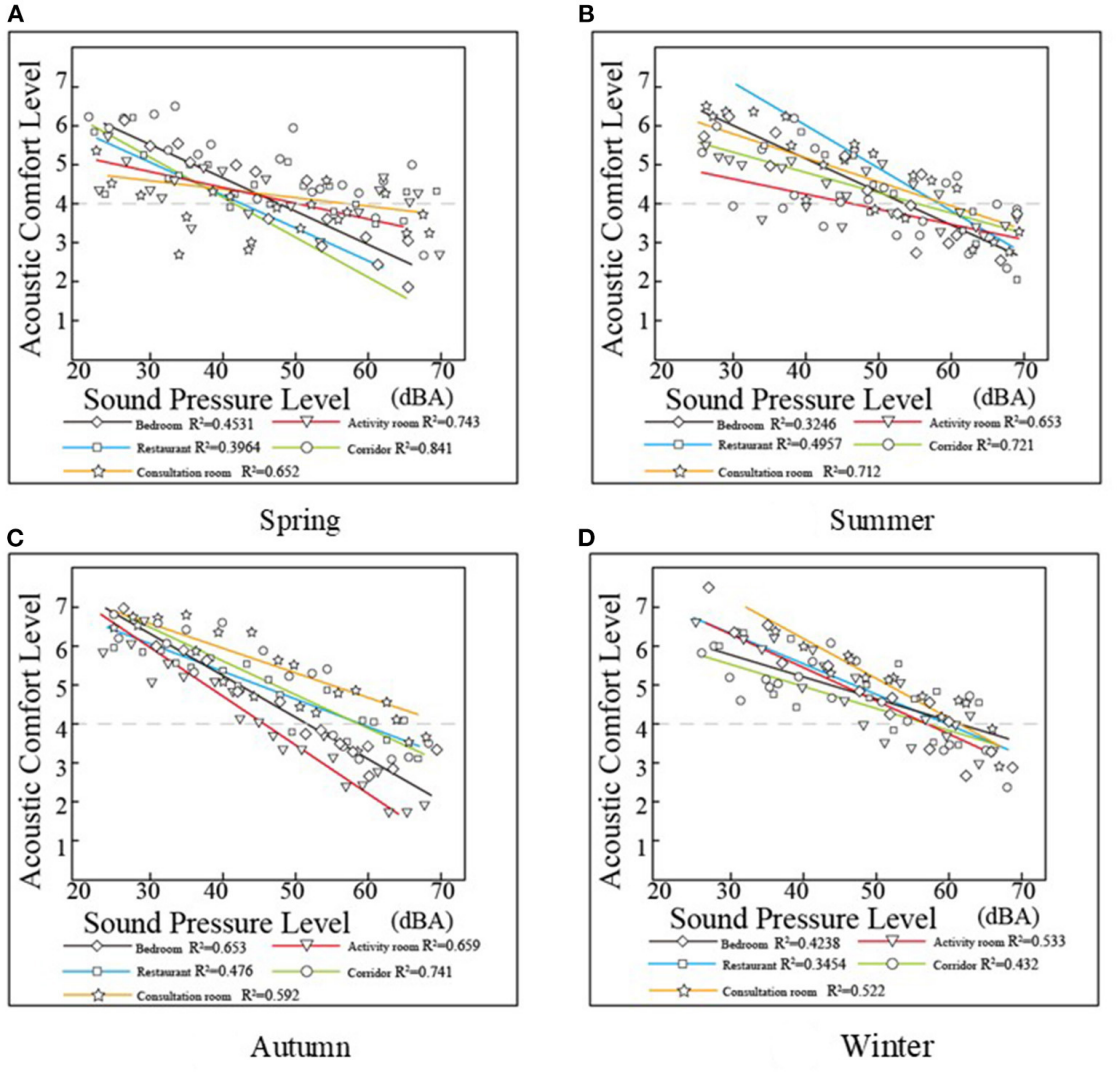
Fitting between SPL and acoustic evaluation based on seasonal differences. **(A)** Spring, **(B)** Summer, **(C)** Autumn, and **(D)** Winter.

The result of the linear relationship between the acoustic evaluation and SPL indicates that regardless of the season or room, the acoustic evaluation decreases as the SPL increases (Figure 4). The linear relationship between the SPL and acoustic evaluation of different types of rooms shows different trends with seasonal changes. The linear relationship between the SPL in the bedroom and restaurant and acoustic evaluation was poor. The linear relationship between corridor SPL and acoustic evaluation was better in spring, summer, and autumn (R-square > 0.7, *p* < 0.01) and worse in winter (R-square = 0.432, *p* < 0.01). The linear relationship between the SPL of the consultation room and acoustic evaluation shows little seasonal variation (R-square between 0.522 and 0.712, *p* < 0.01).

An evaluation value exceeding four points indicates that the participants were satisfied with the surrounding physical environment. In summer, they generally expressed dissatisfaction with the acoustic environment when the SPL exceeded 65 dB(A). The SPL requirements were slightly higher in autumn and winter, and when the level reached 60 dB(A), they expressed dissatisfaction. For bedrooms and activity rooms, the satisfactory level was between 60 dB and 65 dB(A). When the noise level exceeds 70 dB(A), the acceptance of noise is greatly reduced, and the elderly express dissatisfaction (39).

In this study, the acoustic levels in the bedrooms and activity rooms were all higher than the recommended values. The noise generated could originate from floor contact noise (38). Additionally, some staff members occasionally move around and produce noise (38). When most of the elderly were in an activity room or outdoors, the noise level in the bedrooms was reduced. The relationship between the SPL in the bedroom and restaurant and the acoustic evaluation is poor (R-squared < 0.653, *p* < 0.01), and research on the relationship between the SPL in the bedroom and restaurant and the satisfaction of the acoustic environment should be further strengthened to meet the acoustic environment needs of the elderly.

### Evaluation of the Lighting Environment

#### Illuminance and Brightness

**Figure 5:**
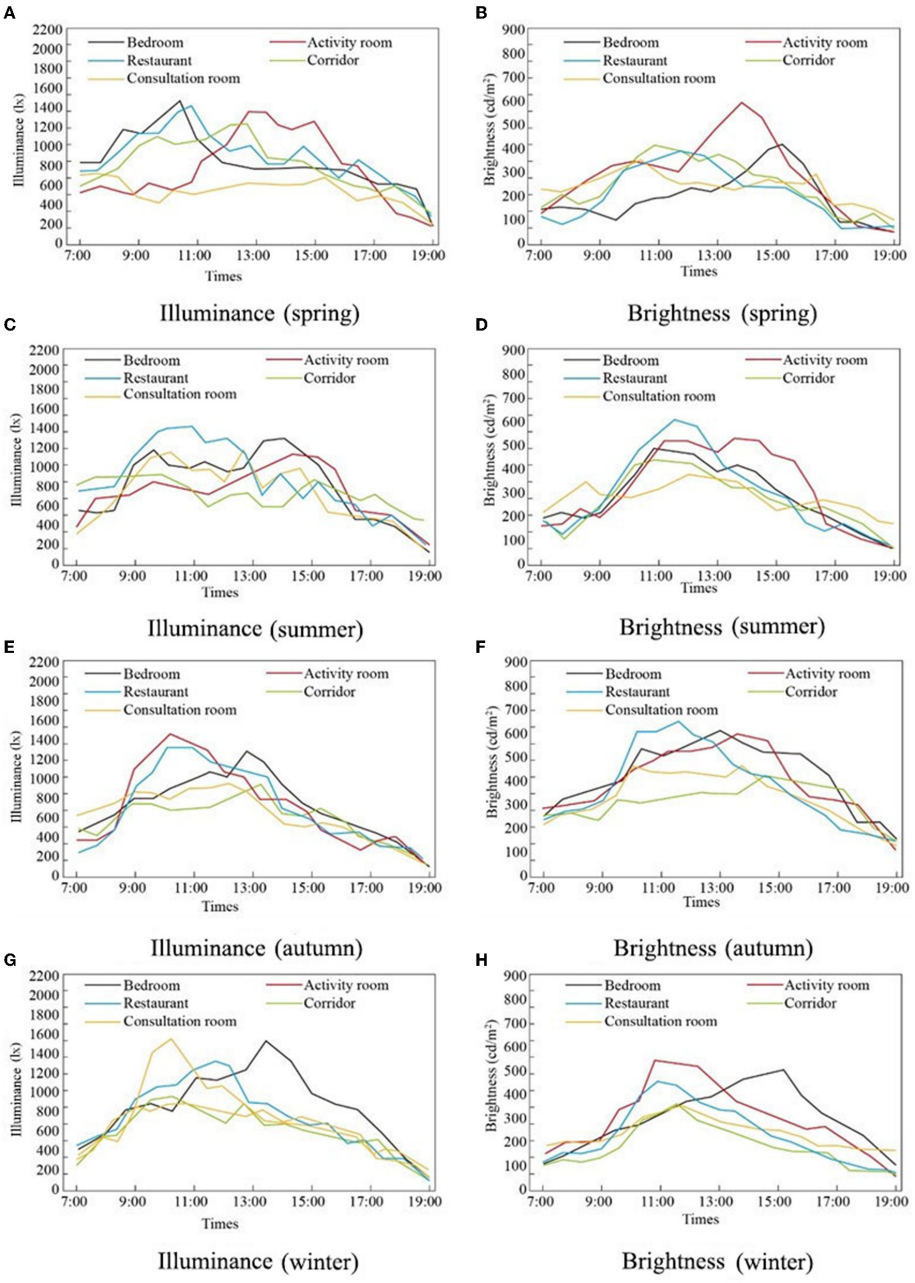
Illuminance and brightness measurement in different seasons and different rooms. **(A)** Illuminance (spring), **(B)** Brightness (spring), **(C)** Illuminance (summer), **(D)** Brightness (summer), **(E)** Illuminance (autumn), **(F)** Brightness (autumn), **(G)** Illuminance (winter), and **(H)** Brightness (winter).

As shown in Figure 5, the illuminance and brightness values of the five types of rooms showed a trend of first increasing and then decreasing, but their respective peaks varied with the seasons. Illuminance peaks in the bedrooms and restaurant occurred between 9:00 and 11:00. The overall brightness in the summer and autumn was higher than that in the spring and winter. According to previous investigations, the activity of the elderly peaks in the morning and before lunch, which was considered in the RECF design. Lighting is usually affected by weather, building location, and orientation. For the elderly who like reading and indoor activities, lighting directly affects their quality of life (40). Seasonal effects also lead to differences in lighting in the RECFs.

The most direct environmental factor affecting the comfort of the elderly is the lighting intensity of the room. According to a Japanese company's research, the overall comfortable illuminance of the room varies from 50 to 250 lx for the elderly; the comfortable illuminance value for younger people accounts for approximately 2/3 of this illuminance, demonstrating that the elderly have a higher demand for lighting (41). In this study, the measured illuminance and brightness were inconsistent. This may be related to the survey season of these RECFs, construction time, structure of the building, and choice of construction materials (42).

#### Evaluation of Lighting Factors

**Figure 6:**
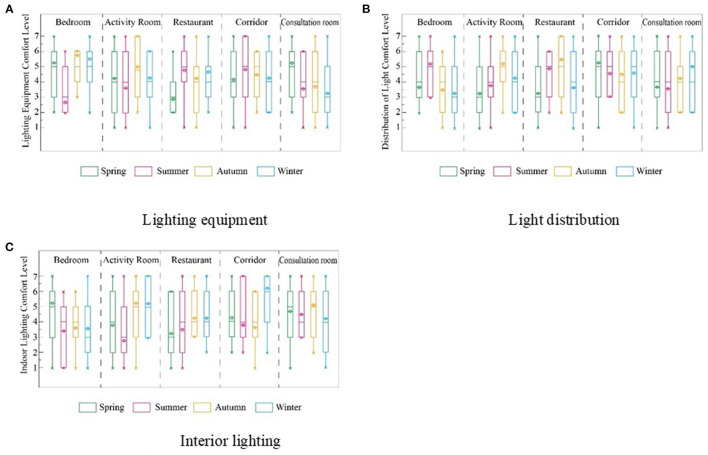
Evaluation of lighting factors of 4 RECFs based on seasonal differences. **(A)** Lighting equipment, **(B)** Light distribution, and **(C)** Interior lighting.

The evaluation of lighting in the bedroom and consultation room was significantly affected by the season (Figure 6). From the perspective of the seasonal changes, it can be seen that the lighting environment evaluation in summer was higher than that in autumn and winter. The lighting environment evaluation was approximately 4.7, 4.0, and 3.9 in summer, winter, and autumn, respectively.

As a high-latitude region, Harbin experiences long days of sunshine in summer, with sufficient sunlight to compensate for the lack of indoor lighting distribution. The average lighting evaluation of the bedroom was 4.5, and the average lighting evaluation of the activity room was 3.5. The evaluation of lighting and lighting distribution in bedrooms was better than that in activity rooms because participants could control the lighting in the bedroom.

Lighting can be adapted to occupants' needs, increasing the happiness index of the elderly and supplementing dim lighting in winter. Mitsuhashi et al. reported the feasibility of using high-intensity artificial light for phototherapy in RECFs and measured the distribution of horizontal illuminance in major public spaces during winter. The horizontal illuminance of the elderly exceeds 2000 lx (43). Changes in vision associated with aging are among the most important physical changes, and the elderly need less lighting than younger adults to perform the same tasks (42, 44). In summer, the corresponding satisfaction is higher because sunlight can supplement lighting. The RECF should strengthen the lighting in spring, autumn, and winter to meet the needs of the elderly for the lighting environment.

#### Relationship Between Lighting Environment and Lighting Evaluation

**Figure 7:**
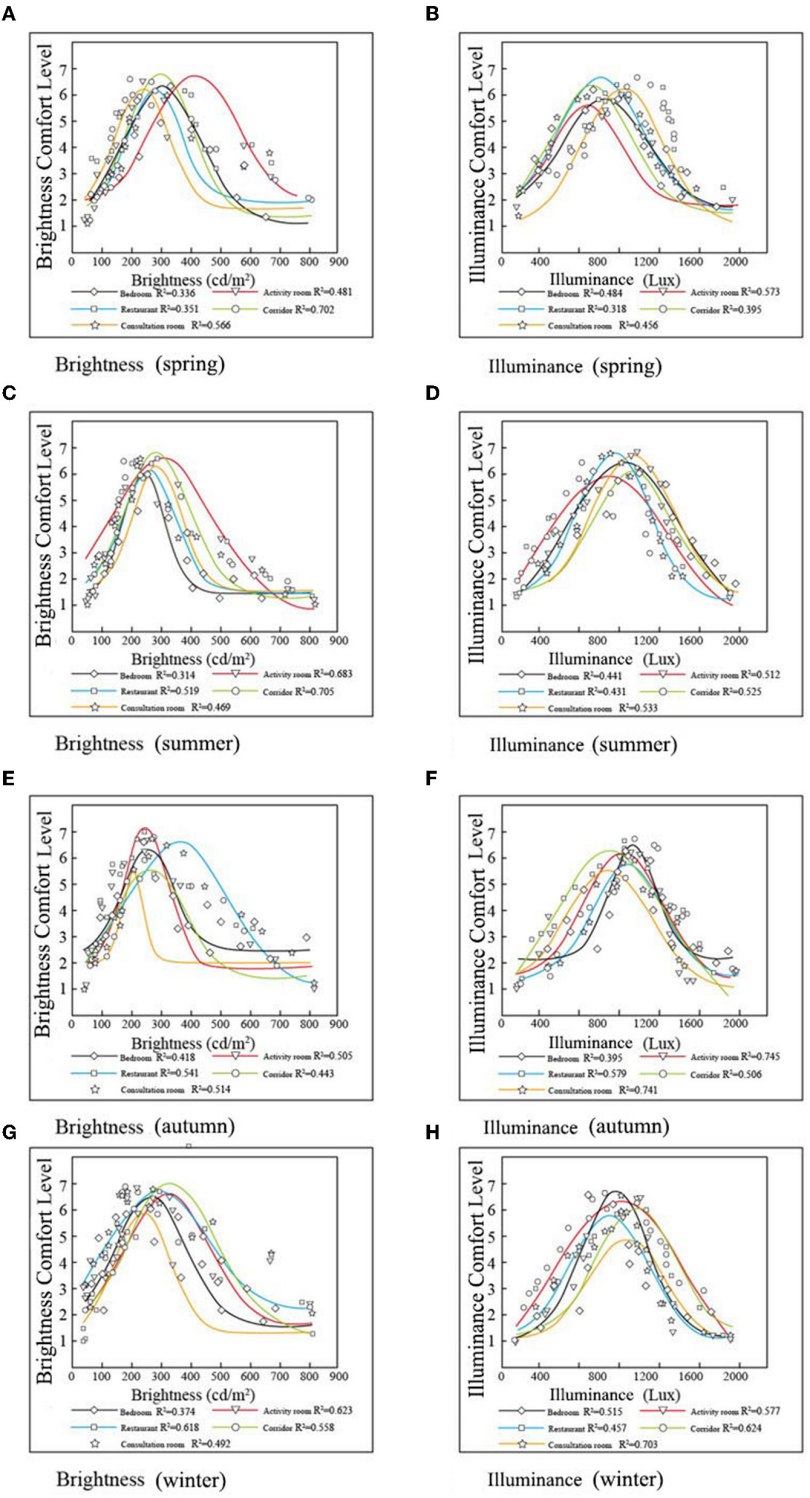
The fit between lighting indicators and lighting evaluation for 4 RECFs. **(A)** Brightness (spring), **(B)** Illuminance (spring), **(C)** Brightness (summer), **(D)** Illuminance (summer), **(E)** Brightness (autumn), **(F)** Illuminance (autumn), **(G)** Brightness (winter), and **(H)** Illuminance (winter).

The lighting indices include brightness and illuminance, which have a nonlinear relationship with the lighting evaluation (Figure 7). It can be seen from the figure that within a certain range, with the increase in brightness and illuminance, the evaluation of brightness and illumination also increases. After a certain limit, with the increase in brightness and illuminance, its evaluation begins to decrease. The linear relationship between bedroom brightness and brightness evaluation was poor (R-squared between 0.314 and 0.418, *p* < 0.01). With changes in spring, summer, autumn, and winter, the linear relationship between the illumination of the consultation room and its evaluation gradually improved (R-squared from 0.456 to 0.703, *p* < 0.01).

The evaluation of the brightness comfort level and illuminance comfort level by the elderly showed significant differences between the measured rooms (*p* < 0.01). The elderly preferred significantly less brightness in autumn and winter, while less illuminance was preferred in spring and summer (Figure 7, *p* < 0.01). The thresholds of the bedrooms and activity rooms were similar, with participants satisfied with an illuminance of <1000 lx. However, in an ordinary room, most of the measured values of vertical and horizontal illuminance were much lower than the reference value of 750 lx, and the illuminance of the corridor was <200 lx (45). The illuminance of the RECFs investigated in this study was higher than the reference value of 750 lx, as indicated by Sinoo in the Netherlands (42). The illuminance and brightness thresholds for the RECFs are not stipulated in official international documents.

In summary, the four RECFs we surveyed met participants' lighting needs. Turner et al. pointed out that insufficient lighting may aggravate insomnia in the elderly population. A lighting threshold of 2,500–3,000 lx has been proven to reduce insomnia in the elderly and improve total sleep time (46). From this perspective, although the main resting space of the elderly is the bedroom, the lighting in ordinary rooms is also critical to the daily activities of the elderly. The corridor was the center of each room. If lighting conditions are poor, the elderly may be in danger of falling. Therefore, RECF should consider the lighting conditions of corridors and other areas that are usually overlooked.

### Evaluation of the Thermal Environment

#### Temperature and Humidity

**Figure 8:**
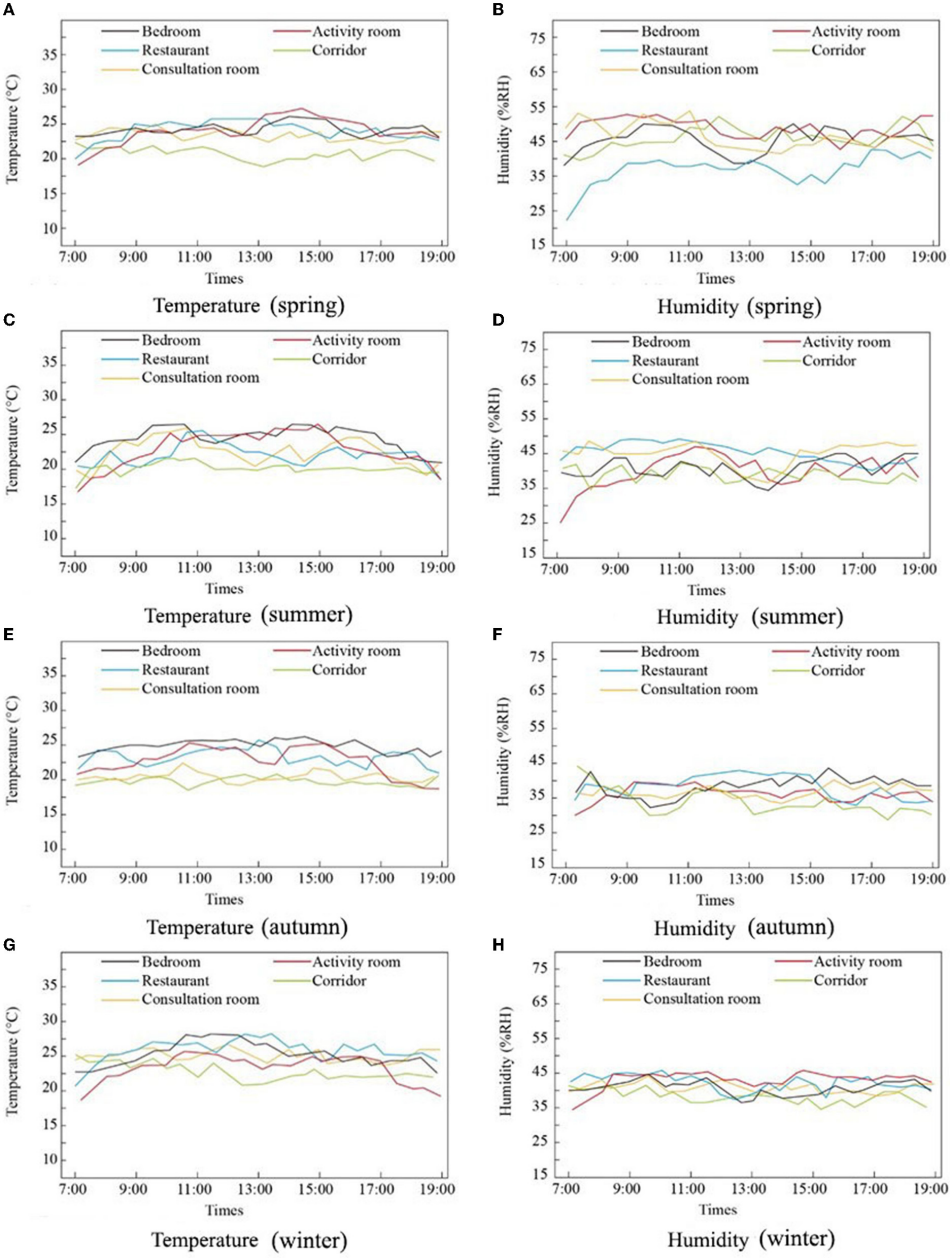
Temperature and Humidity measurement in different seasons and different rooms. **(A)** Temperature (spring), **(B)** Humidity (spring), **(C)** Temperature (summer), **(D)** Humidity (summer), **(E)** Temperature (autumn), **(F)** Humidity (autumn), **(G)** Temperature (winter), and **(H)** Humidity (winter).

As shown in Figure 8, the temperature fluctuations of the five types of rooms during the four seasons were small, indicating that the RECFs selected in this study were better for indoor temperature control.

Temperature and humidity are key indicators for assessing room comfort. The temperature measurements showed no obvious differences between the active rooms and bedrooms. In line with the findings of some previous studies (47), our research found that the humidity in the activity rooms was higher than that in the bedrooms.

Maintaining air comfort in RECFs is important, but requires consideration of the activities of the elderly. Related research has indicated that in the absence of physical activity, the indoor temperature should be close to 25°C; in the case of physical activity, it should be lowered but remain higher than 20°C. Humidity should be maintained between 25 and 55% (48). Additionally, according to T18883–2002, the standard temperatures for heating in winter should fall within 16–24°C and for air conditioning in summer should be 22–28°C (49). The temperatures in this study were either higher or lower than the recommended temperatures in summer and winter.

#### Evaluation of Thermal Environment Factors

**Figure 9:**
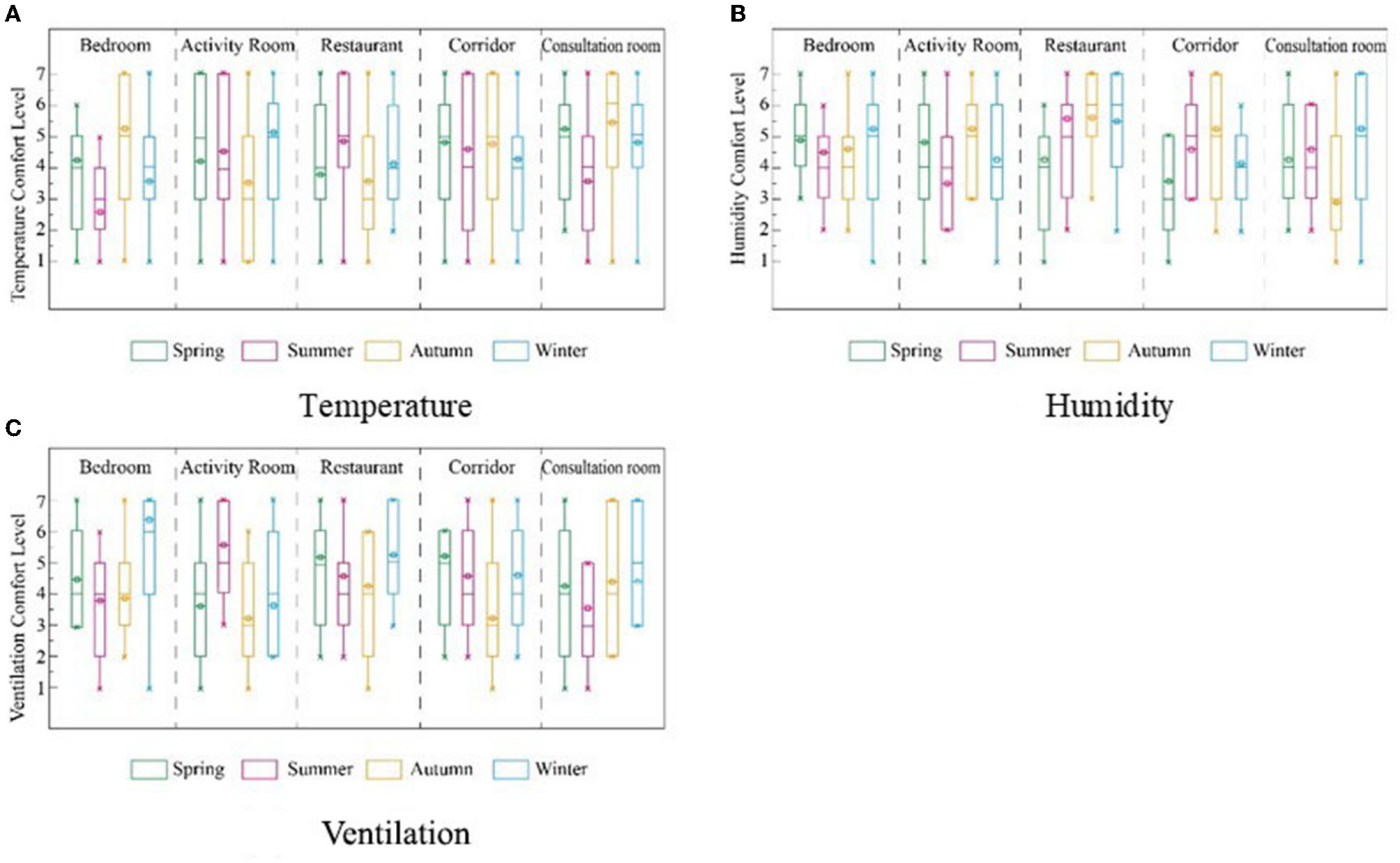
Evaluation of thermal factors of 4 RECFs based on seasonal differences. **(A)** Temperature, **(B)** Humidity, and **(C)** Ventilation.

Our research analyzed three thermal environment indicators (temperature, relative humidity, and ventilation) of different RECFs and their correlation with the IEQ (Figure 9). The results demonstrate that the elderly were satisfied with their RECF thermal environment, and almost all the temperature and ventilation evaluations exceeded four points.

The temperature evaluation of the bedroom, corridor, and consultation room showed a similar trend to seasonal changes; that is, the evaluation in summer was significantly lower than that in spring, autumn, and winter. The temperature evaluation of active rooms and restaurants showed a similar change trend with seasons; that is, the evaluation in summer was higher than that in spring and autumn (Figure 9A).

The humidity evaluation of the five types of rooms showed no obvious regularity with season (Figure 9B). As the elderly can often open windows or doors at will, the bedroom ventilation is highly rated (Figure 9C). Because ventilation in activity rooms cannot meet everyone's needs, there are differences between the RECFs. Generally, however, they evaluated the ventilation in autumn as low. The analysis of the amount of ventilation and humidification required in winter indicated that the variety of ventilation methods of each facility, such as opening windows and living room doors, is closely related to building performance: the colder the area, the more the ventilation increases (50). Overall, the elderly in this study had higher satisfaction with the thermal environment of RECF. Further research should focus on ventilation in activity rooms.

#### Relationship Between Thermal Environment and Thermal Evaluation

**Figure 10:**
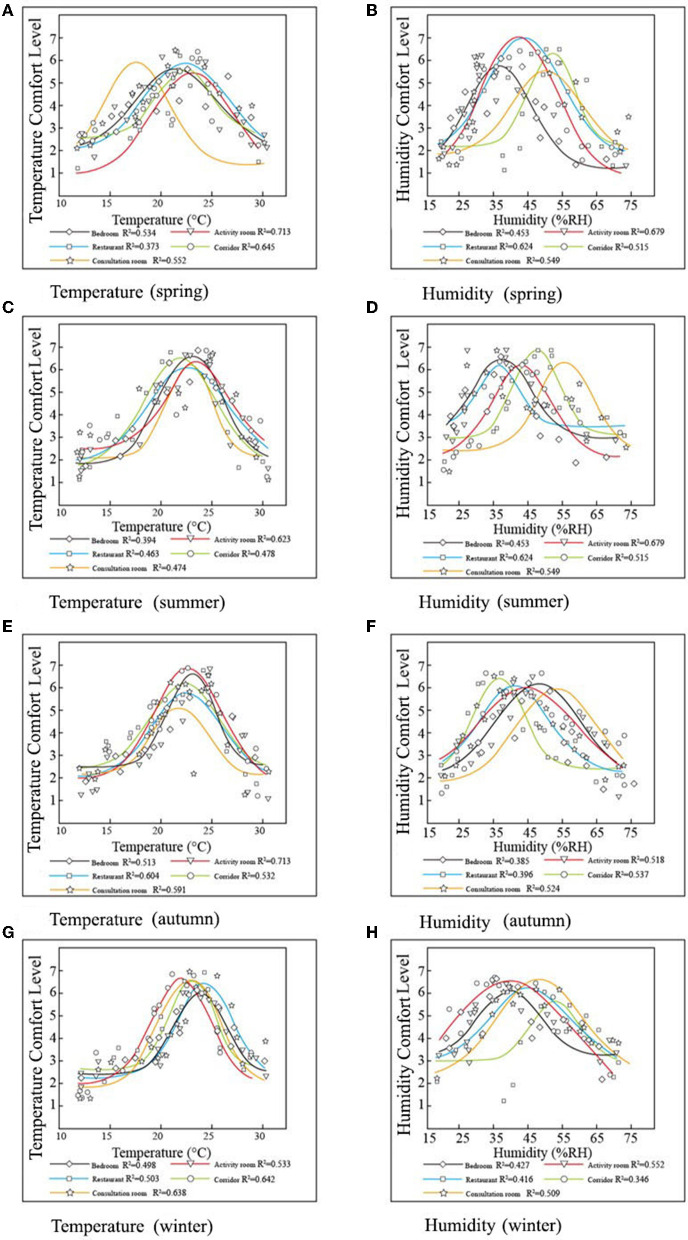
The fit between thermal indicators and thermal evaluation for 4 RECFs. **(A)** Temperature (spring), **(B)** Humidity (spring), **(C)** Temperature (summer), **(D)** Humidity (summer), **(E)** Temperature (autumn), **(F)** Humidity (autumn), **(G)** Temperature (winter), and **(H)** Humidity (winter).

There was a good fit between the seasonal temperature and thermal environment evaluation (Figure 10). For all the RECFs, the threshold did not vary greatly depending on the season or room, and the elderly were satisfied with temperatures between 25 and 26.5°C. Regardless of room or season, a humidity of 40–45% was most comfortable for the elderly. There were significant differences between the evaluation values of temperature and humidity in the five rooms (*p* < 0.01).

Unlike the results of Taleghani et al. and Jin et al. (51, 52), our results indicate that the elderly prefer a drier environment, with some interviewees stating that high humidity makes them feel breathless, hot, and stuffy.

Physically and psychologically, elderly prefer warm environments. For sedentary elderly individuals, the best temperature is approximately 25.3°C. The temperature range is 16–25°C in winter and 22–31°C in summer (53). The satisfaction of the elderly with temperature and humidity in summer is lower than that in other seasons, indicating that the temperature and humidity control of RECF in Northeast China is still lacking in terms of seasonal replacement.

### Evaluation of Indoor Air Quality (IAQ)

**Figure 11:**
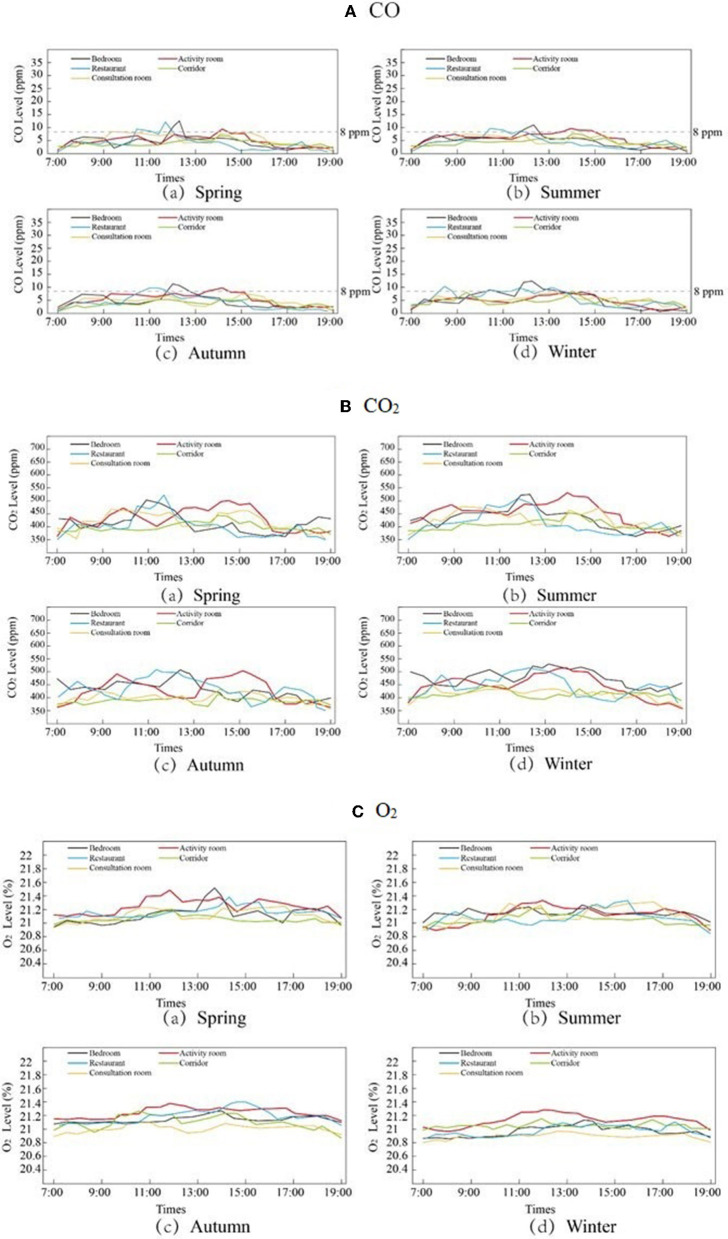
CO CO_2_ and O_2_ measurement in different seasons and different rooms. **(A)** CO, **(B)** CO_2_, and **(C)** O_2_.

As shown in Figure 11, the CO levels in all five rooms were relatively stable. The CO levels were below 8 ppm in all four seasons, meeting the national safety standards. The CO_2_ content in the different types of rooms showed different trends. The CO_2_ levels in the corridors and consultation rooms varied less over the time of the day (approximately 400 ppm) and were stable across seasons. The CO_2_ concentration in the bedroom and restaurant generally peaks at 11:00–13:00, whereas the peak CO_2_ concentration in the activity room generally occurs between 9:00 and 10:00 in the morning and between 14:00 and 16:00 in the afternoon (Figure 11). This finding is consistent with the life and rest rules of the elderly population. The oxygen content of the five types of rooms was relatively stable. In the four seasons, the oxygen content fluctuated around 21%, which was in line with the normal standard.

**Figure 12:**
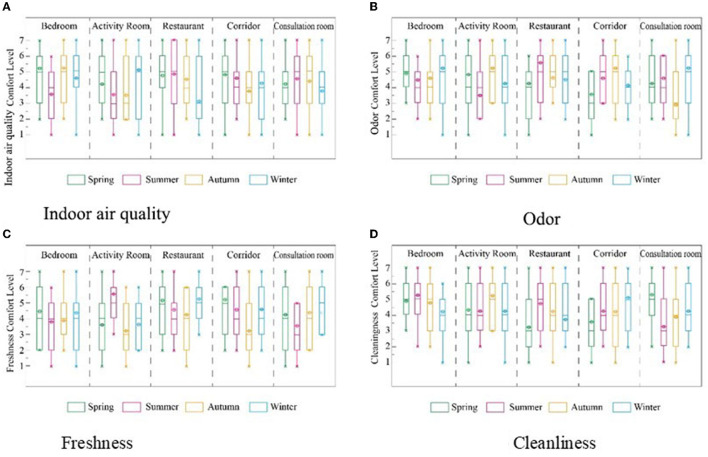
Evaluation of air quality of 4 RECFs based on seasonal differences. **(A)** Indoor air quality, **(B)** Odor, **(C)** Freshness, and **(D)** Cleanliness.

As people spend more than 90% of their time indoors, IAQ is important (54). In the survey, IAQ evaluation included odor, freshness, and cleanliness (Figure 12). As a result, the elderly are less sensitive to odors and correlate poorly with overall IEQ assessments, even though living environment odors are critical to the health of the elderly. Research on IAQ in facilities for the elderly has an important impact on their quality of life and health. Mendes et al. evaluated IAQ in the urban elderly care center (ECC) of 425 elderly individuals in Porto, Portugal, and found that fungal concentrations often exceeded reference levels. In addition, other pollutants exceeded the reference levels (55). Bedrooms and playrooms are places where seniors often spend their time. Moreover, the activity room is relatively crowded, and the IAQ is worse. Therefore, targeted IAQ improvement measures should be implemented for different types of rooms.

### Relationship Between Physical Environmental Factors and Overall IEQ

#### Correlation Between IEQ Indicators Evaluation and Physical Environment

To study the influence of physical environment on the overall IEQ evaluation, we first analyzed the correlation between different physical environments and the overall IEQ (see Table 3). The elderly had a good overall IEQ evaluation of the four physical environmental parameters, all of which exceeded 4.5 points (out of seven). The four selected physical environment parameters had a high positive correlation with the overall IEQ evaluation and a strong influence on IEQ, with all correlation coefficients close to or above 0.7 (*p* < 0.001; see Table 3).

**Table 3:** Correlations between IEQ and four environmental parameters.

**Overall IEQ**	**Acoustic**	**Lighting**	**Thermal-Env**	**IAQ**
Evaluation	4.64	4.52	5.27	4.76
Correlation coefficient	0.730	0.686	0.731	0.691
*p*-value	0.001^***^	0.001^***^	0.001^***^	0.001^***^

*** *p < 0.001*.

The four IEQ factors were further studied to evaluate the physical environmental parameters of the RECFs comprehensively (Table 4). In Table 4, column A shows the mean evaluation and standard deviation of the four factors of the elderly in different types of rooms.

**Table 4:** Evaluation of four RECFs.

	**Factors**	**Mean subjective evaluations**	**Correlation coefficient**
Activity room	Acoustic	4.59/1.845	0.717/0.000 (^***^)
	Lighting	4.13/2.042	0.705/0.000 (^***^)
	Thermal-Env	5.21/1.916	0.732/0.000 (^***^)
	IAQ	4.31/2.004	0.736/0.000 (^***^)
Bedroom	Acoustic	3.82/1.794	0.695/0.000 (^***^)
	Lighting	3.96/1.965	0.740/0.000 (^***^)
	Thermal-Env	4.67/1.959	0.849/0.000 (^***^)
	IAQ	4.12/2.060	0.715/0.000 (^***^)
Restaurant	Acoustic	5.28/1.992	0.784/0.000 (^***^)
	Lighting	4.83/1.988	0.666/0.000 (^***^)
	Thermal-Env	4.81/1.747	0.694/0.000 (^***^)
	IAQ	5.16/1.812	0.708/0.000 (^***^)
Corridor	Acoustic	3.65/1.798	0.826/0.000 (^***^)
	Lighting	3.40/1.946	0.701/0.000 (^***^)
	Thermal-Env	4.96/1.935	0.849/0.000 (^***^)
	IAQ	3.45/1.837	0.664/0.000 (^***^)
Consultation room	Acoustic	3.45/1.783	0.674/0.000 (^***^)
	Lighting	4.76/1.953	0.726/0.000 (^***^)
	Thermal-Env	5.20/1.895	0.851/0.000 (^***^)
	IAQ	4.57/1.864	0.658/0.000 (^***^)

*** *p < 0.001*.

Activity rooms and restaurants had the highest ratings, with close to five points. For corridor, the scores for acoustics, lighting, and IAQ were lower than four points. The lower corridor evaluation (from Column B) might be due to poor acoustic and lighting environments because their correlation with IEQ evaluation was as high as 0.826 and 0.701 (*p* < 0.001). The IEQ evaluations of the other five types of rooms have a high correlation with the four physical parameters, with correlation coefficients of between 0.66–0.85 (*p* < 0.001).

#### Evaluation of IEQ Factors by Season and Location

This study found that seasonal changes affected IEQ satisfaction. Autumn had the highest IEQ evaluation, with all four factors being close to five points (*p* < 0.01). Participants generally indicated that spring and autumn were more comfortable than summer and winter (see Table 5), perhaps because the elderly prefer the climate in spring and autumn. Particularly in the IAQ evaluation, the spring (5.183) and autumn (5.071) evaluations were significantly higher than the summer (4.179) and winter (4.113) evaluations (*p* < 0.01). The reason for the high IEQ satisfaction during autumn might be the suitable thermal environment in autumn, warming in spring, and the end of the hot summer in autumn. However, the frequent opening of windows in the summer can cause outdoor traffic noise and air pollution. The abundance of sunshine in summer may be pleasant for the elderly, but too bright a light can affect their actions. However, lack of sunlight in winter can also affect the activities of the elderly (56). For example, Sinoo et al. found that in seven RECFs, at least three-quarters of the measurements had corridor brightness significantly below the 200 lx threshold (42). Therefore, in winter, or when there is insufficient lighting, the RECF lighting environment should be considered. Mishima et al. also demonstrated that we need to consider the elderly who suffer from poor environmental lighting, leading to disorganized circadian rhythms (57). Therefore, in winter, or when there is insufficient lighting, the RECF lighting environment should be considered. For different room types, the bedrooms were more comfortable than activity rooms based on the evaluation, perhaps because bedrooms are private spaces and residents have more freedom to open windows, close doors, or pull curtains according to their preferences. The relative advantages and costs of private bedrooms are controversial, especially given the current commitment to creating cost-effective and people-centered care facilities. Similar to our results, Calkins et al. also found that bedrooms are better than shared activity rooms in RECFs (58).

**Table 5:** Evaluations of IEQ factors based on seasonal differences and location differences.

	**Factors**	**Acoustic**	**Lighting**	**Thermal-Environment**	**IAQ**
Seasonal factors	Spring	4.514	4.207	4.158	5.183
	Summer	4.979	3.687	3.987	4.179
	Autumn	4.409	4.909	4.955	5.071
	Winter	3.459	3.946	4.188	4.113
	F	32.732	34.923	24.794	22.746
	P	0.003	0.001	0.005	0.000
	$\eta_{\text{p}}^{\text{2}}$	0.069	0.073	0.053	0.049
Location factors	Bedroom	4.391	4.877	4.803	5.140
	Activity room	4.613	4.540	4.798	4.708
	Restaurant	4.802	3.937	3.753	4.275
	Corridor	3.767	4.163	4.562	4.302
	Consultation room	5.228	3.895	5.014	4.395
	F	37.732	35.483	32.504	38.415
	P	0.000	0.001	0.000	0.002
	$\eta_{\text{p}}^{\text{2}}$	0.146	0.139	0.129	0.149

### Effects of Demographic Factors

IEQ satisfaction has a significant relationship with people's background (59–63). This background includes physiological factors such as gender and age; social factors such as education, retirement, and marital status; and lifestyle factors such as residence time and place of origin. This section includes an analysis of the impact of these factors on the IEQ satisfaction of the elderly based on all RECF surveys. Seven personal and social factors that may affect IEQ satisfaction (Table 6) were selected. In general, gender differences had no significant impact on IEQ factor evaluation, while residence time and marital status had an impact (*p* < 0.01). Age and education had a significant effect on all IEQ factors except temperature (*p* < 0.01). The following subsections discuss the impact of personal and social factors on IEQ evaluation.

**Table 6:** Relationship between IEQ evaluations value and residents' background.

		**Acoustics**	**Lighting**	**Temperature**	**Humidity**	**Odor**	**IAQ**
Gender	Male	4.71	4.6	5.34	5.11	4.83	4.83
	Female	4.34	4.22	5.12	4.62	4.52	4.5
	F	4.945	5.076	3.927	9.879	6.932	5.395
	P	0.345	0.939	0.531	0.424	0.352	0.693
	$\eta_{\text{p}}^{\text{2}}$	3.661	4.154	2.141	5.718	4.715	3.885
Age	<65	5.12	5	4.92	3.04	3.14	5.16
	65–75	4.13	3.98	4.94	4.71	4.56	4.37
	75–85	4.24	4.21	5.06	5.24	5.04	4.54
	>85	5.31	5.54	5.04	3	2.08	6.32
	F	9.848	9.301	1.18	34.711	25.278	6.949
	P	0	0	0.086	0	0	0
	$\eta_{\text{p}}^{\text{2}}$	3.554	4.036	1.147	4.232	4.355	3.804
Education	Basic-Edu	3.56	3.43	5.07	4.8	4.58	3.68
	Secondary-Edu	4.55	4.53	4.98	4.28	4.25	4.82
	Higher-Edu	4.61	4.53	4.74	5.19	4.94	4.99
	F	28.965	30.405	2.579	12.699	6.706	39.926
	P	0	0	0.077	0	0.001	0
	$\eta_{\text{p}}^{\text{2}}$	3.443	3.89	2.119	4.594	4.654	3.567
Pension	<2K	2.52	2.51	4.64	5.54	5.52	2.61
	2–5K	5.03	4.92	4.94	4.22	4.12	5.22
	>5K	4.84	5.03	4.92	4.73	4.34	5.24
	F	109.697	109.458	1.234	17.677	28.344	101.611
	P	0	0	0.094	0	0	0
	$\eta_{\text{p}}^{\text{2}}$	2.938	3.332	2.145	4.544	4.439	3.161
Residence time	<1 year	3.92	3.82	4.84	4.51	4.21	4
	1–3 years	4.22	4.21	4.86	4.83	4.74	4.52
	3–5 years	4.86	4.83	5.13	4.93	4.92	5.21
	>5 years	5.31	5.12	4.55	4	4.04	6.1
	F	13.398	13.103	4.741	13.96	14.586	27.762
	P	0	0	0.04	0	0	0
	$\eta_{\text{p}}^{\text{2}}$	3.513	3.985	3.142	4.517	4.506	5.672
Former residence	Countryside	4.23	4.12	3.92	3.64	3.47	4.44
	Country town	4.05	4.05	4.65	3.84	3.76	4.20
	Suburb	3.94	3.74	4.78	4.72	4.64	4.23
	City	4.63	4.53	5.02	5.54	5.46	4.96
	F	1.942	2.619	64.626	80.480	84.905	4.974
	P	0.164	0.106	0.000	0.010	0.000	0.026
	$\eta_{\text{p}}^{\text{2}}$	3.593	4.055	1.176	4.156	4.171	3.790
Marital status	Single	5.52	5.42	4.02	2.45	2.23	5.93
	Married	4.83	4.75	4.95	3.94	3.84	5.1
	Widowed	3.04	2.94	4.68	5.24	5.01	3.13
	Divorced	4.53	4.63	5.02	6.17	5.62	4.86
	F	12.106	9.307	13.903	58.512	53.677	14.945
	P	0.001	0.002	0	0	0	0
	$\eta_{\text{p}}^{\text{2}}$	3	3.454	1.245	3.959	4.008	3.19

*Basic-Edu includes primary school or lower; Secondary-Edu includes junior or senior school; Higher-Edu includes college or higher education*.

### Influence of Physiological Factors on the Evaluation of IEQ Factors

**Figure 13:**
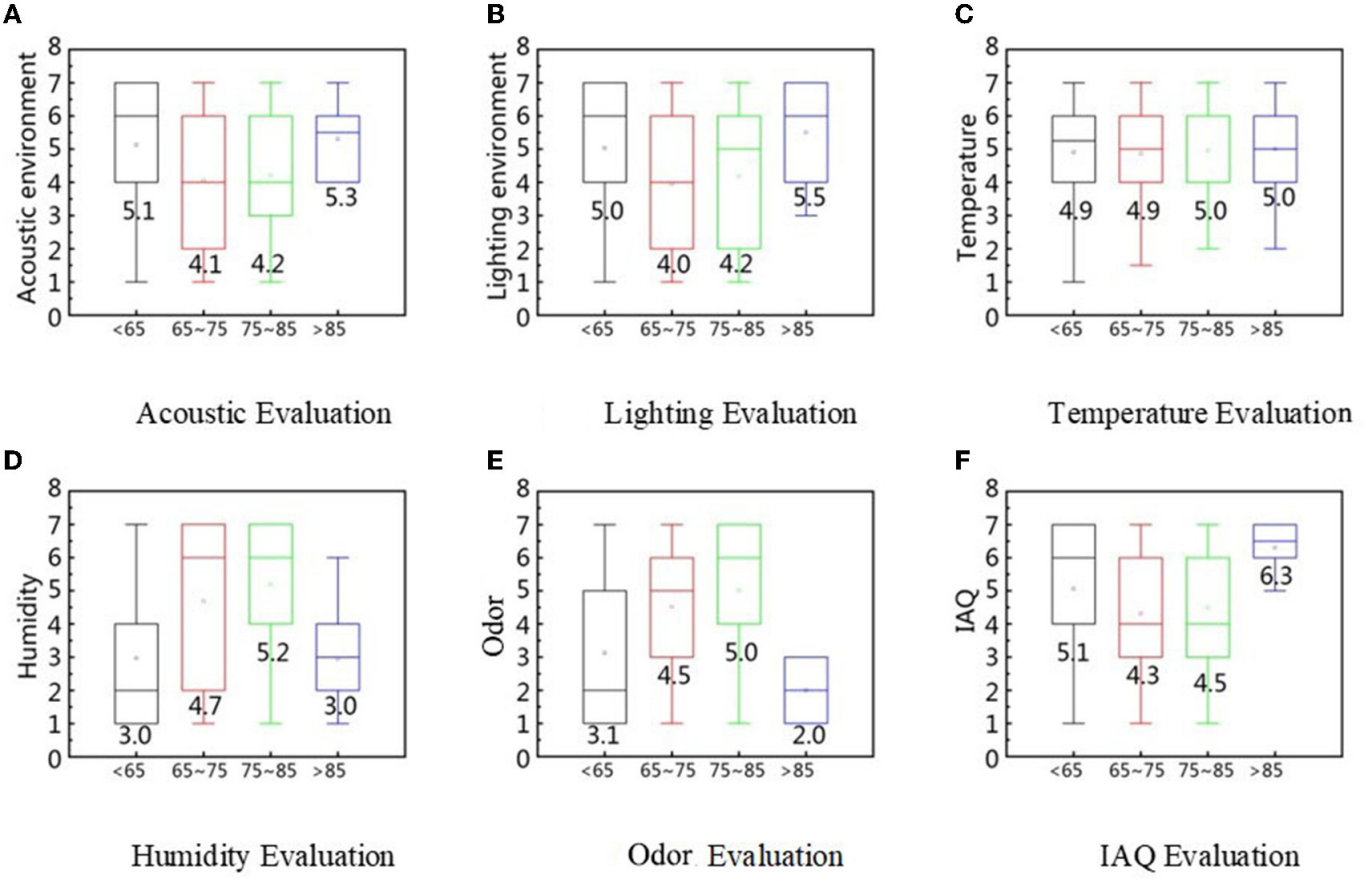
IEQ factors evaluation based on age difference. **(A)** Acoustic evaluation, **(B)** Lighting evaluation, **(C)** Temperature evaluation, **(D)** Humidity evaluation, **(E)** Odor evaluation, and **(F)** IAQ evaluation.

This study explored the effects of different age groups on lighting, acoustics, and IAQ. The evaluations of the under-65 and over-85 groups were similar, with ratings of the lighting environment, acoustic environment, and IAQ higher. However, the two 65–85-year groups rated humidity evaluation and odor evaluation higher, perhaps because those over 85 have poor physical functioning and relatively low sensitivity to the environment, making it difficult for them to perceive changes. In addition, some studies have shown that with an increase in age, the evaluation of the comfort of the acoustic environment of the elderly is increasing, indicating that the tolerance of the elderly to the acoustic environment is higher than that of the young (64). In our study, the 66–85-year age group also rated slightly lower than those aged 60–65 years (see Figure 13). As the light received by the retina decreases with age, the elderly require more lighting. After the age of 70 years, they have difficulty seeing some details, vision loss, and the lighting requirements increase after the age of 50 (44). Although most studies suggest that as the visual function of the elderly declines, lighting conditions have a greater impact on them. However, the study by Wang et al. showed that there were no significant differences in the subjective evaluations and ECG indicators of the elderly under different lighting conditions (65). The influence of lighting color on the elderly has gradually attracted attention. Studies have found that low-saturation lighting can help improve the mood and comfort of the elderly, and medium-high saturation lighting can affect thermal sensations in the elderly (66). Therefore, the RECF under investigation still needs to improve the lighting conditions. According to Indraganti and Peng's research results, there is no obvious correlation between age and thermal comfort, and gender and age have a minimal influence on thermal evaluation (67, 68). However, we found that the elderly were more sensitive to changes in humidity. This result is consistent with the findings of Jin et al. (69).

### Influence of Social Factors on the Evaluation of IEQ Factors

**Figure 14:**
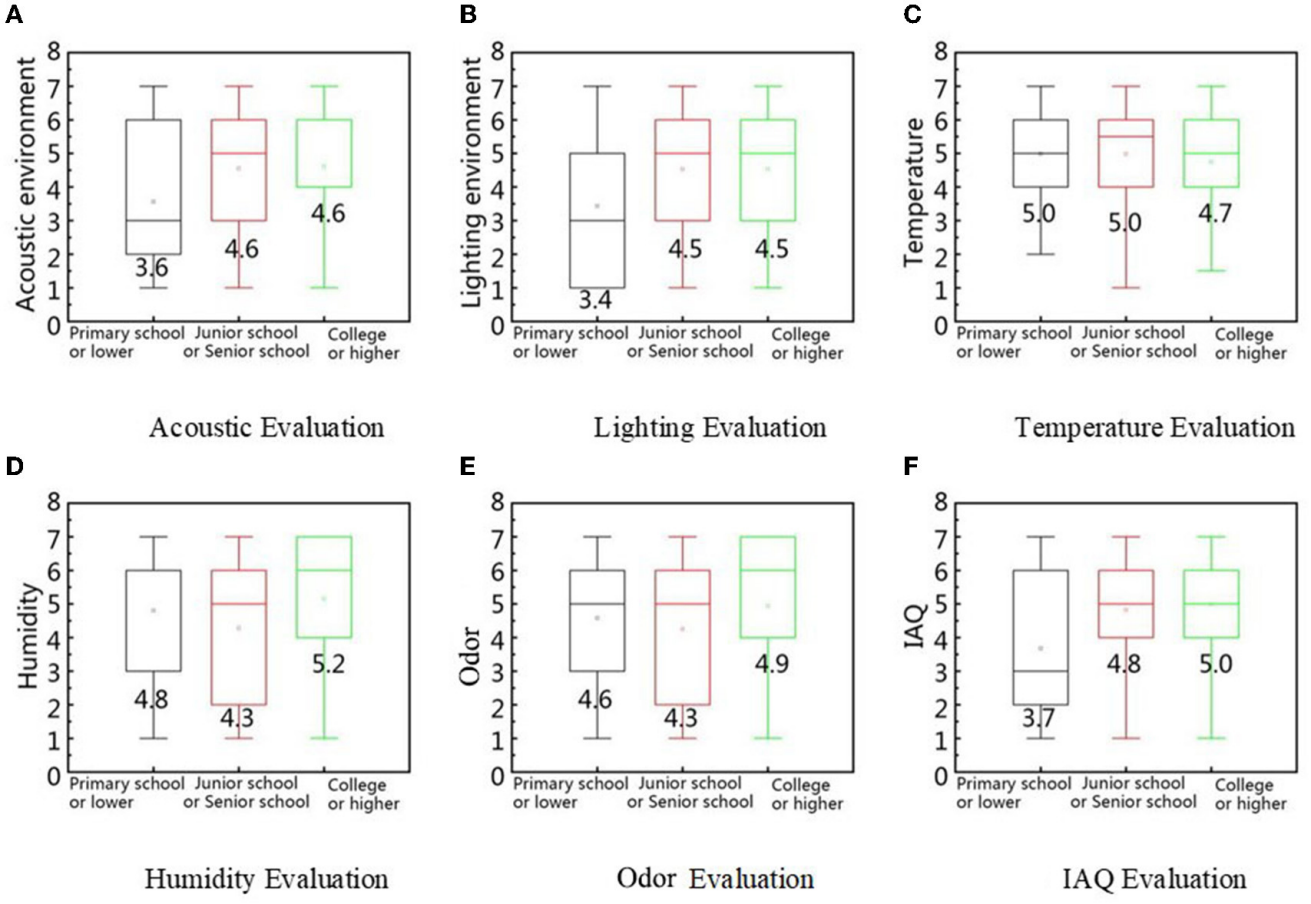
IEQ factors evaluation based on the difference in education level. **(A)** Acoustic evaluation, **(B)** Lighting evaluation, **(C)** Temperature evaluation, **(D)** Humidity evaluation, **(E)** Odor evaluation, and **(F)** IAQ evaluation.

As shown in Figure 14, in a quiet environment, people with higher education and pension levels are more comfortable. By contrast, in a noisy environment, acoustic comfort decreases with an improvement in education level. Elementary school or below is classified as basic education, junior high school or high school as secondary education, and college degree or above as higher education. The boundary between quiet and noisy environments is an SPL of 60 dB(A) (70). The elderly with higher education had higher personal attainment and usually gave positive evaluations, whereas those with basic education had relatively low IAQ ratings (below four points). The evaluation of the elderly with secondary and higher education was higher than that of those with a basic education (*p* < 0.01). The elderly with basic education had relatively low IAQ scores (below four points). According to a survey by Guo et al., this difference comes from the respondents' different understandings of IAQ (71). They found that people with higher cultural backgrounds had higher evaluations of IAQ.

**Figure 15:**
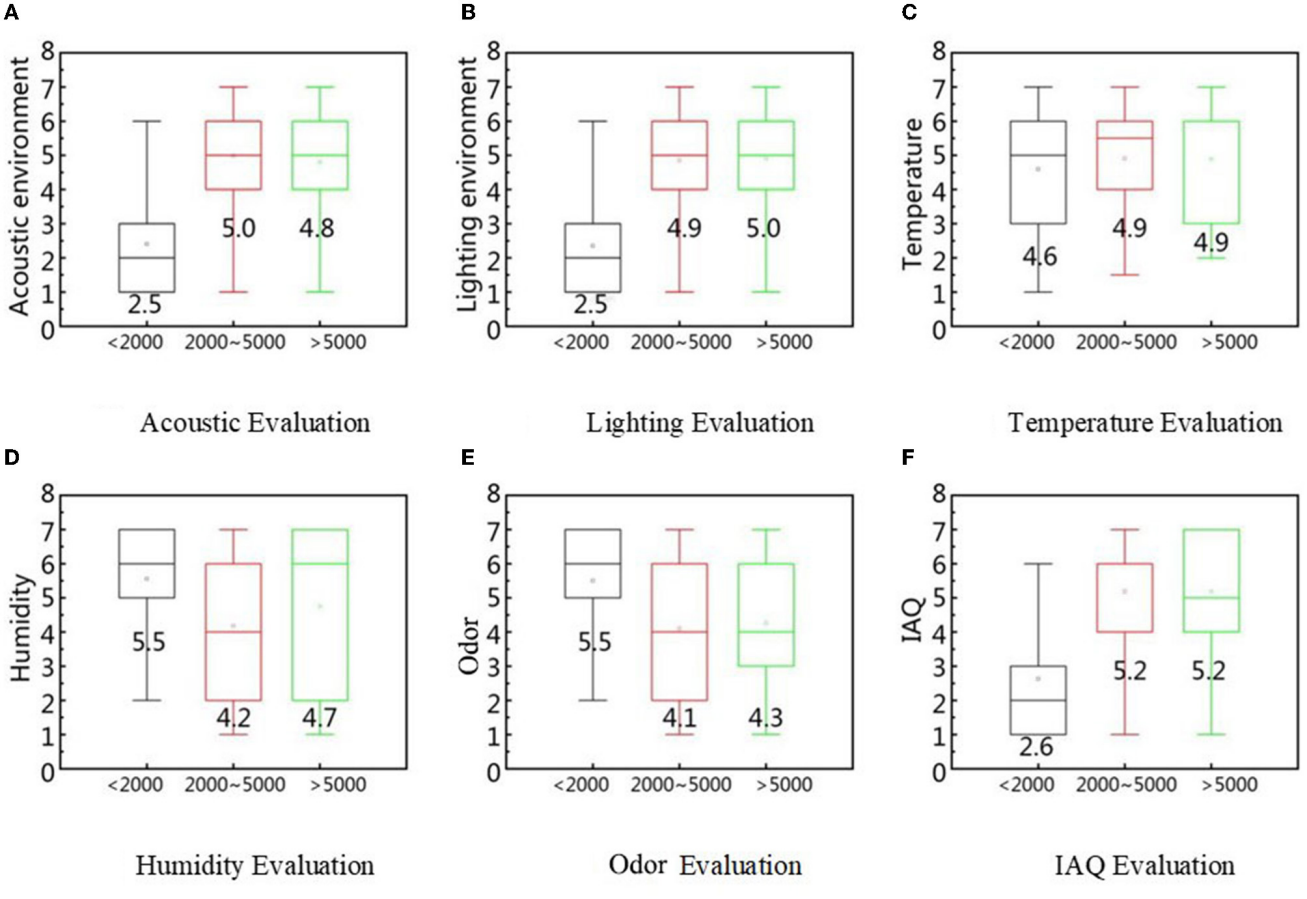
IEQ factors evaluation based on pension difference. **(A)** Acoustic evaluation, **(B)** Lighting evaluation, **(C)** Temperature evaluation, **(D)** Humidity evaluation, **(E)** Odor evaluation, and **(F)** IAQ evaluation.

The impact of pensions on the evaluation results was similar to that of education level (Figure 15). This is likely because basic education corresponds to a low pension (<2,000), and higher education to a high pension (>5,000). Elderly people with low pensions and basic education have stricter environmental requirements, and those with higher pensions and higher education have a higher tolerance for the environment. Similar to the present study, Cui et al. showed that with an increase in income, the elderly's evaluation of acoustic environment comfort decreases (64).

Marital status can cause large differences in IEQ satisfaction (Supplementary Figure 6). Overall, divorced older adults had a higher IEQ rating than other groups; widowed older adults had an average IEQ rating of less than four points, and married older adults had a relatively stable evaluation of various factors, unlike the single group. However, in contrast to the findings of Cui et al. (64), our results demonstrate that the divorced elderly were more satisfied with the environment than the married elderly (*p* < 0.01), perhaps because the former are more likely to be emotionally suffering; the RECF environment increases their socialization, making up for the lack of a close relationship, greatly alleviating their cognitive impairment. Håkansson et al. demonstrated that unmarried (single, divorced, and widowed) people are more likely to have cognitive impairment than married people (72), which can create uncertainty in evaluating the surrounding environment.

Moreover, according to research by MOUSAVI-NASAB and others, married (and divorced) people will have strong episodic memory, which is good for the health of the elderly, while single and widowed people will have negative memory and cognition, impacting them in the long run (73). Therefore, in the IEQ, the scores of divorced and married older adults were higher than those of widowed and single individuals (*p* < 0.01).

### Influence of Lifestyle Factors on Evaluation of IEQ Factors

The residence time also had an impact on IEQ evaluation. With increased time spent in RECFs, the IEQ evaluation also increased (Supplementary Figure 7). The elderly who had lived in RECFs for >3 years had an average evaluation score of five points, especially for the acoustic environment, lighting environment, and IAQ. Simultaneously, their long residence times indicated their satisfaction with the RECF environment. The satisfaction of the elderly with the IEQ increases with their residence time, perhaps because they are accustomed to living there. If they are more familiar with the living environment, they will have a higher evaluation of it (74).

## Conclusion

The physical environments of the four RECFs were found to be highly correlated with IEQ evaluation, including acoustic, lighting, thermal environment, and IAQ (*p* < 0.01). The survey found that IEQ evaluations were affected by seasonality, with the evaluation of bedrooms being higher than that of activity rooms. A reasonable assessment of the surveyed RECF was moderate, while the elderly had a weaker understanding of intelligibility. The acoustic evaluation has a linear relationship with SPL and is not affected by the season or room. The lighting environment in summer is usually higher than that in winter and autumn, and the bedroom evaluation is usually higher than that of the activity room. Brightness and illuminance have a nonlinear relationship with lighting evaluation. The relative humidity of the different types of rooms varied greatly in spring and less in winter. IEQ satisfaction of all four RECFs was highly correlated with four physical parameters, with correlation coefficients of 0.66–0.85 (*p* < 0.001). Seasonal changes affected IEQ satisfaction, which was highest in autumn. The overall evaluation results demonstrated that in all four RECFs, bedrooms were more comfortable than activity rooms. It also indicates that the evaluation of the elderly of the RECF is not affected by gender; the effect of age is reflected in environmental factors other than temperature, and people under 65 and over 85 have the largest fluctuations in physical environment evaluation. Additionally, the elderly were satisfied with the overall IEQ environment of the RECFs.

This study investigated the environment of elderly care facilities where the elderly live, which will help improve the living environment of the elderly in the future. To the best of our knowledge, there is a lack of research on the living environment of RECFs, and the true scale of the problem is yet to be evaluated. We provide the first step toward bridging this gap.

This study summarized four RECF environments in the northeast region, representing the global cold zone from summer to winter in 2018. Statistical analyses were conducted on these data using specific site parameters and surveys of relevant elderly individuals. However, their satisfaction was still low, which may explain their unclear description, or it may be just abnormal data. These problems should be addressed in future research by exploring the potential impacts or mediating factors between various physical environmental evaluations.

In the future, the proportion of the elderly in China will further increase, and the elderly will have higher requirements for the indoor environment. The results of this study can be used to guide the renovation of existing facilities and the design of interior spaces in new ones. From the perspective of the impact of the acoustic environment, lighting environment, thermal environment, and IAQ on the elderly, the elderly care environment in Northeast China should be optimized. In addition, the elderly may have different environmental needs for different types of rooms. For example, in the bedroom, acoustic and lighting environments interact more closely with the health status of the elderly. In an activity room, IAQ may be a more important factor for the elderly. Therefore, research on the interaction between the indoor environment and the health and condition of the elderly is very important to improve their lives.

## Data Availability Statement

The original contributions presented in the study are included in the article/Supplementary Material, further inquiries can be directed to the corresponding author.

## Author Contributions

JM and JK contributed to conception and design of the study and wrote sections of the manuscript. JM organized the database, performed the statistical analysis, and wrote the first draft of the manuscript. Both authors contributed to manuscript revision, read, and approved the submitted version.

## Funding

This work was supported by the National Natural Science Foundation of China (NSFC; grant number: 51778169), China Association for Science and Technology Think Tank Young Talents Program 2021 (grant number: 2021ZZZLFZB1207149), and Ministry of Science and Technology of China (grant number: G2021179030L).

## Conflict of Interest

The authors declare that the research was conducted in the absence of any commercial or financial relationships that could be construed as a potential conflict of interest.

## Publisher's Note

All claims expressed in this article are solely those of the authors and do not necessarily represent those of their affiliated organizations, or those of the publisher, the editors and the reviewers. Any product that may be evaluated in this article, or claim that may be made by its manufacturer, is not guaranteed or endorsed by the publisher.
