# Supplementary material for: Indoor Environmental Quality of Residential Elderly Care Facilities in Northeast China
Source: Front Public Health. 2022 May 4;10:860976. doi: 10.3389/fpubh.2022.860976 (PMC9116475; doi:10.3389/fpubh.2022.860976)
Supplement: Supplementary file 1 [file Table_1.DOCX]

Supplementary table 1. Questionnaire and scales.

| Category | Questions | Scale |
| --- | --- | --- |
| Background information | Gender, age, education level, pension, marriage, place of residence, and length of stay |  |
| Subject satisfaction | Satisfaction with acoustic  Satisfaction with visual  Thermal comfort  Satisfaction with IAQ  Overall comfort | Very dissatisfied (1 point) to very satisfied (7 points) |
