# Supplementary figures and images for: Indoor Environmental Quality of Residential Elderly Care Facilities in Northeast China
Source: Front Public Health. 2022 May 4;10:860976. doi: 10.3389/fpubh.2022.860976 (PMC9116475; doi:10.3389/fpubh.2022.860976)

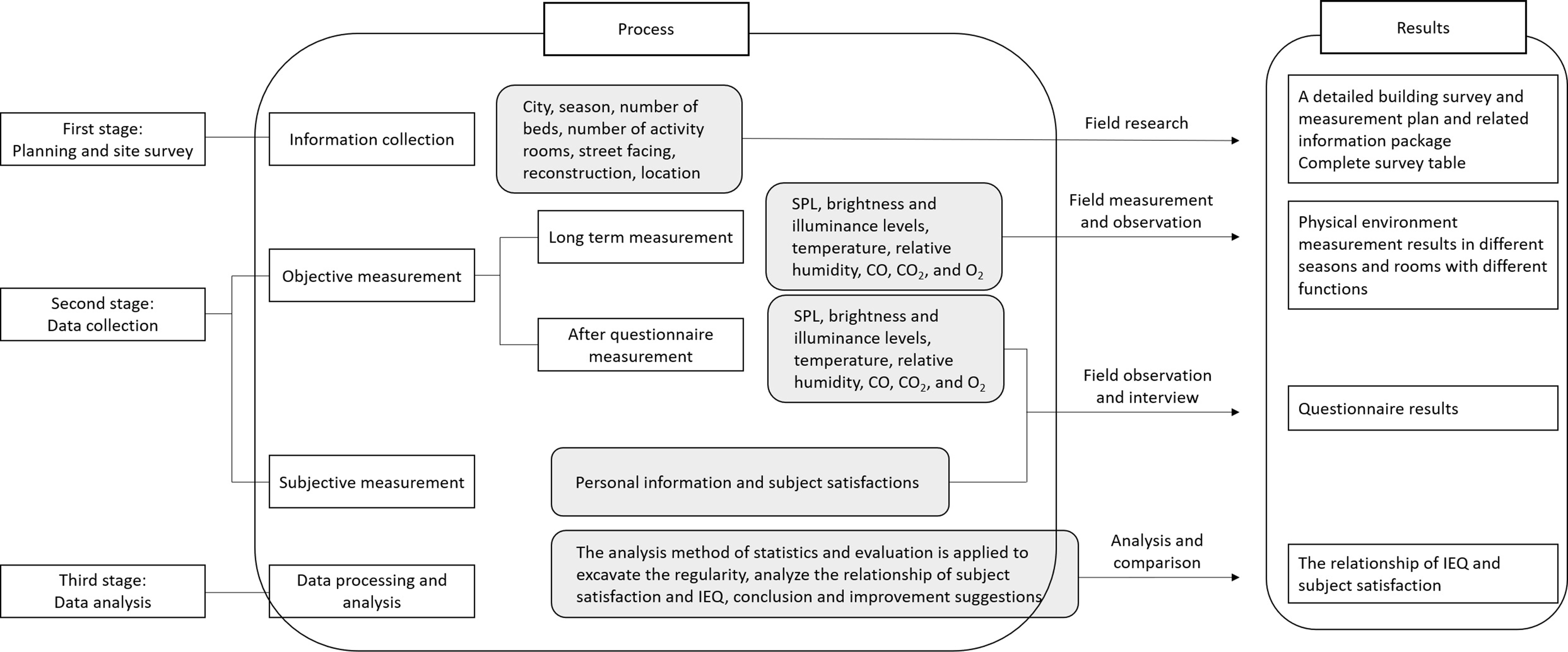

Supplement: Supplementary file 2 [file Image_1.JPEG]

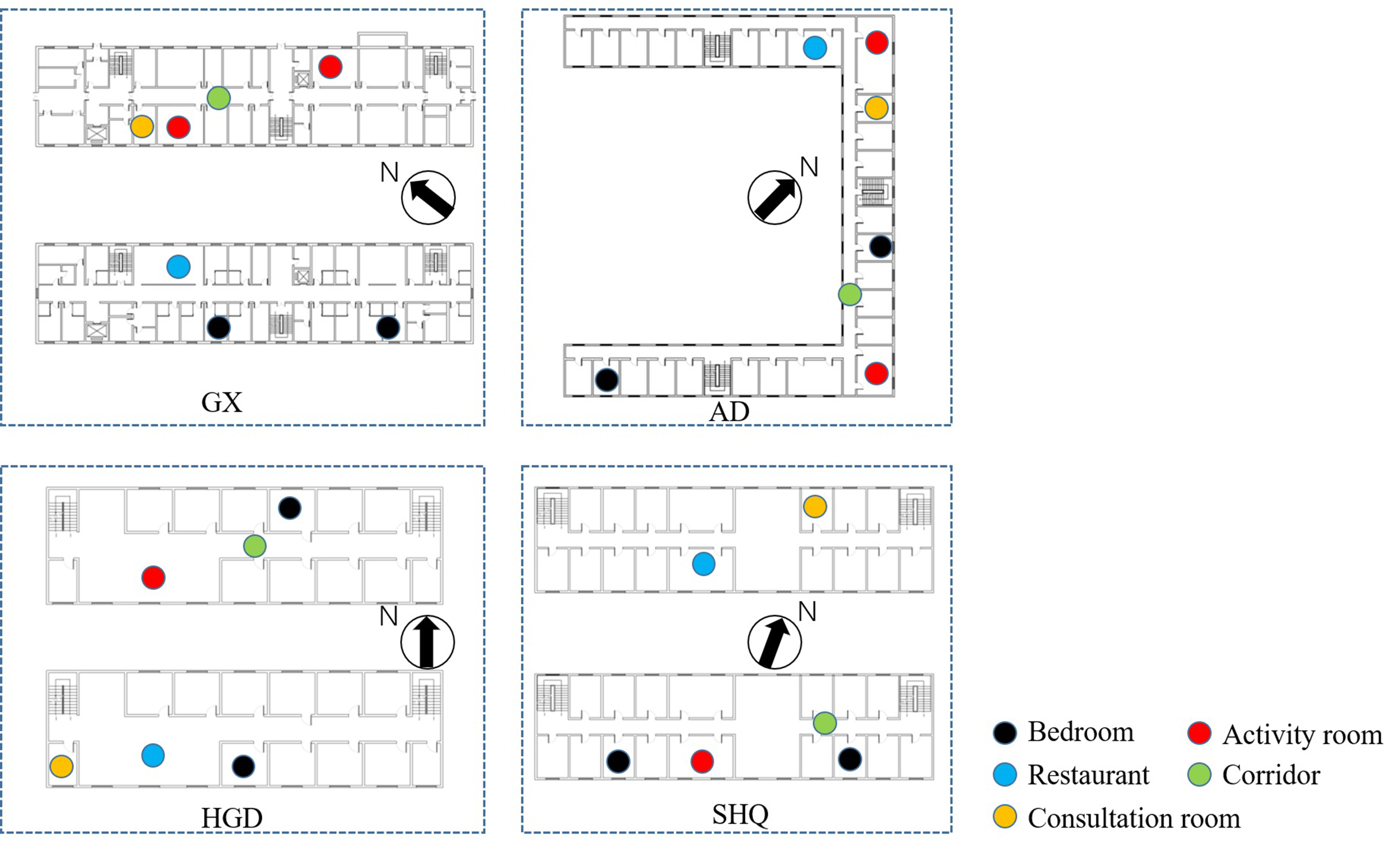

Supplement: Supplementary file 3 [file Image_2.JPEG]

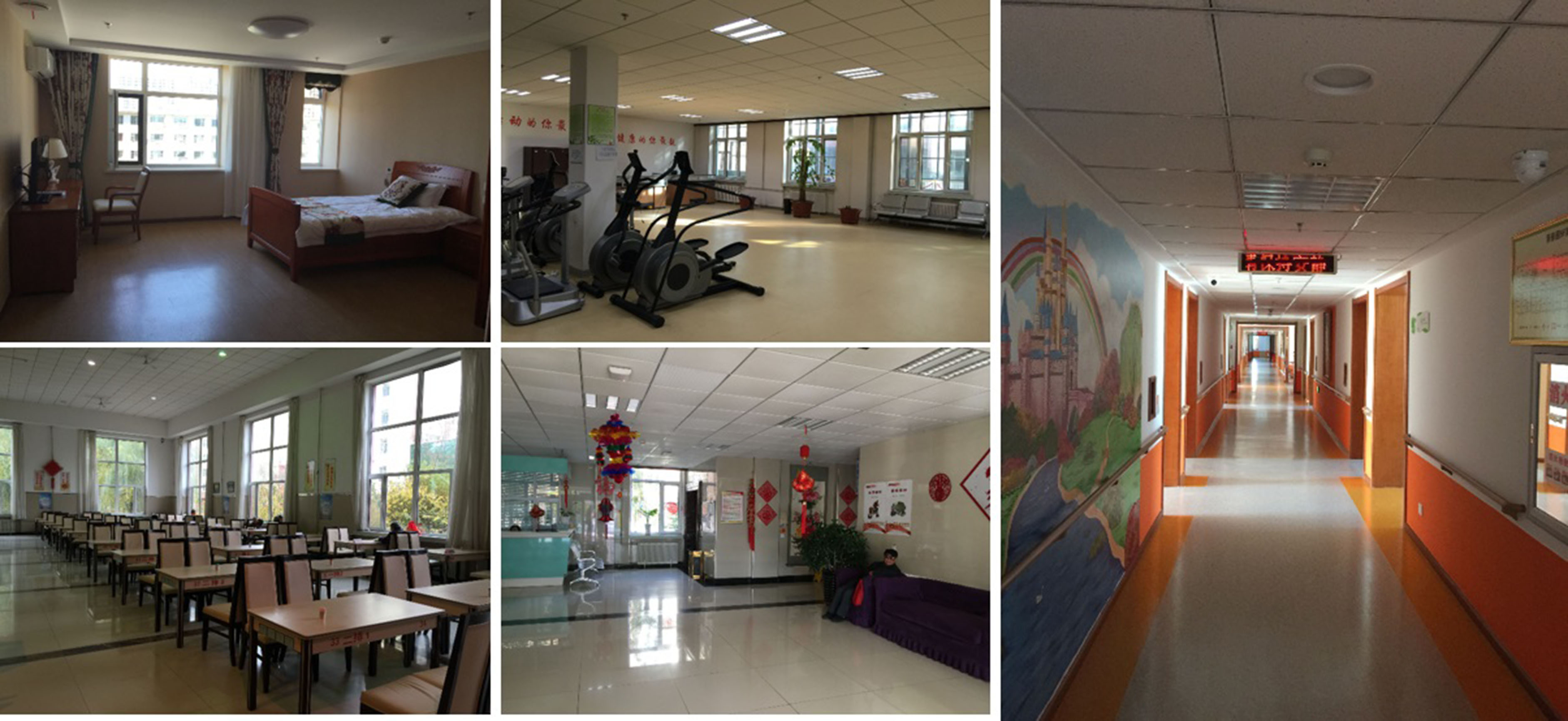

Supplement: Supplementary file 4 [file Image_3.JPEG]

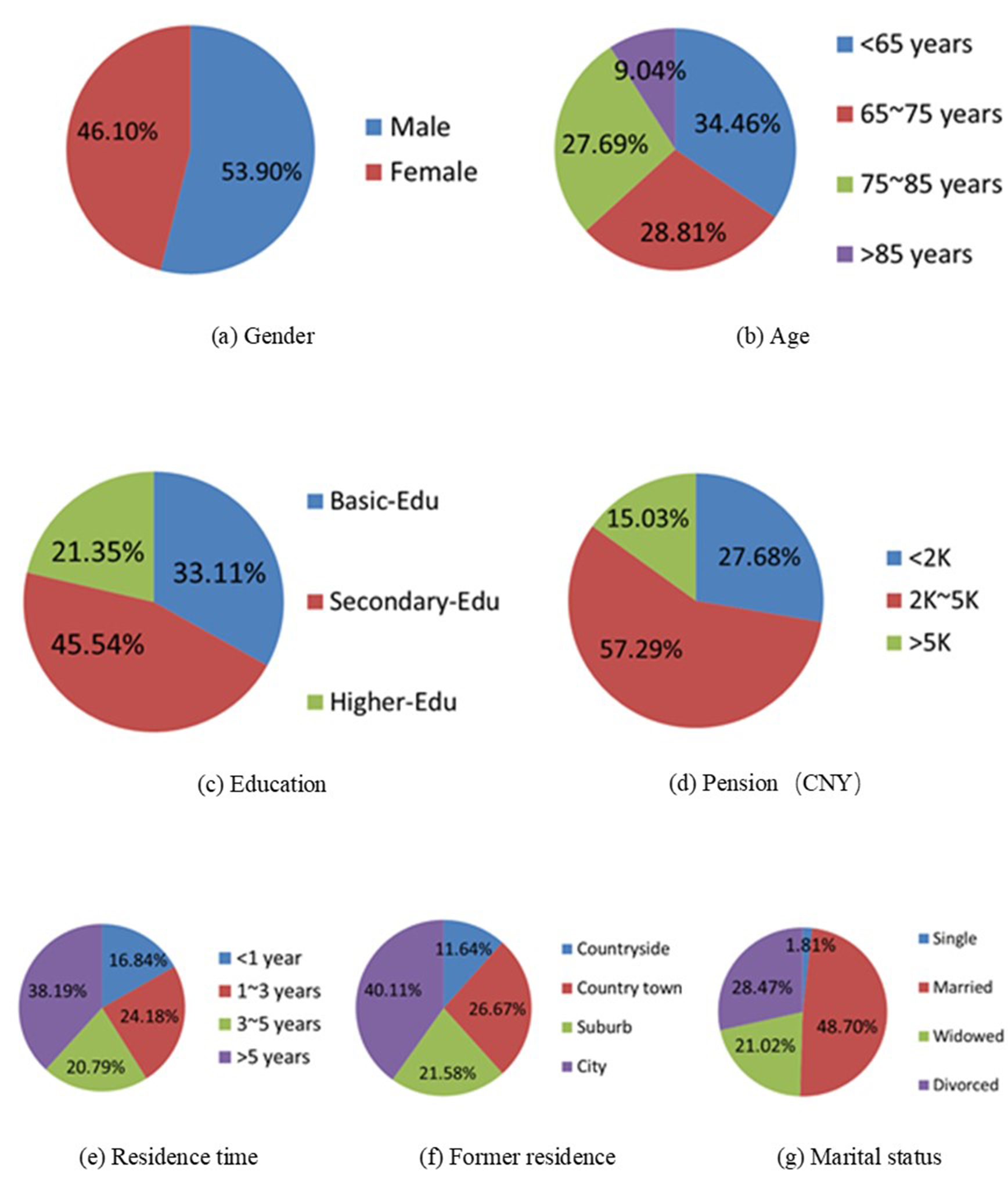

Supplement: Supplementary file 5 [file Image_4.JPEG]

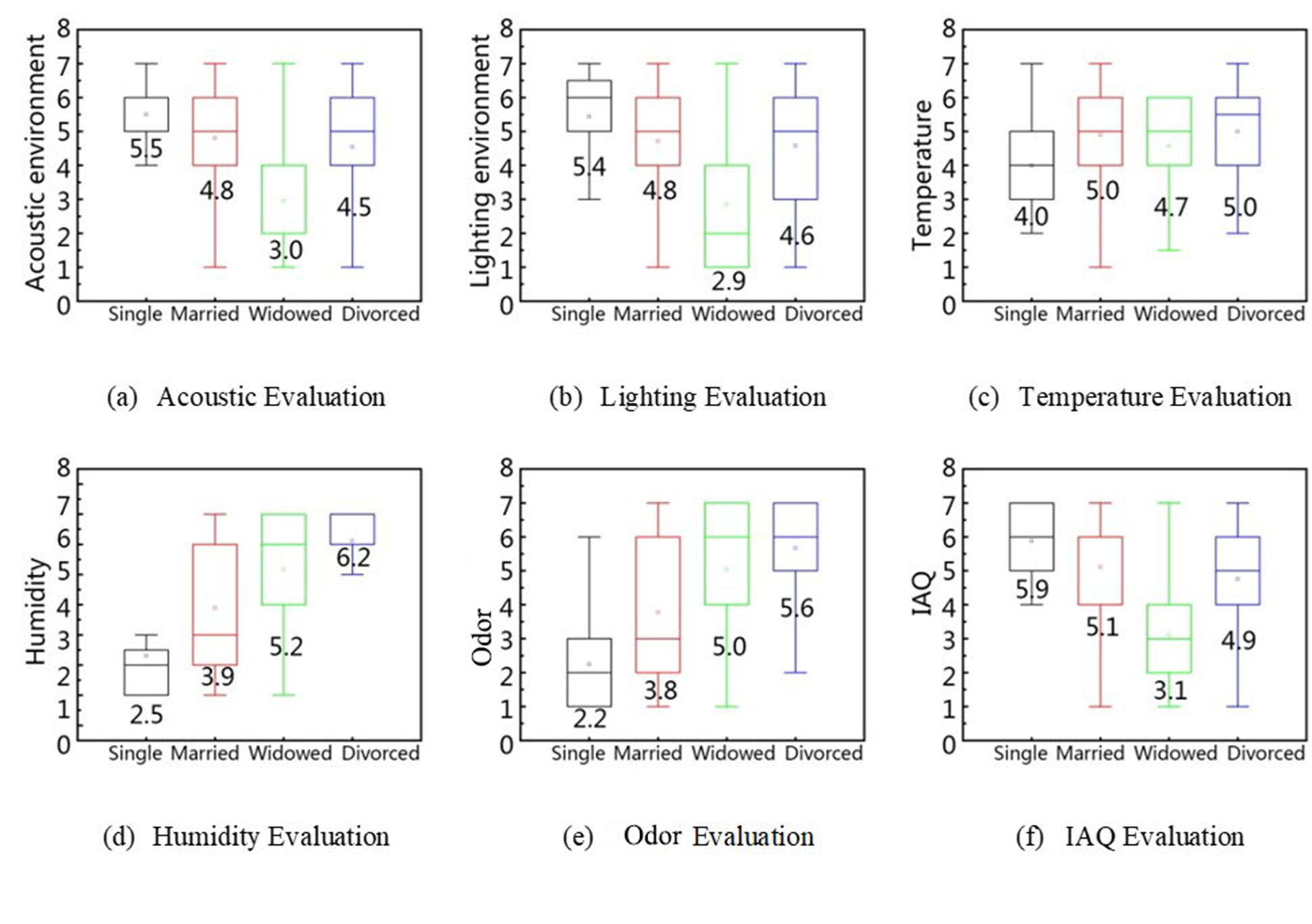

Supplement: Supplementary file 7 [file Image_6.jpg]

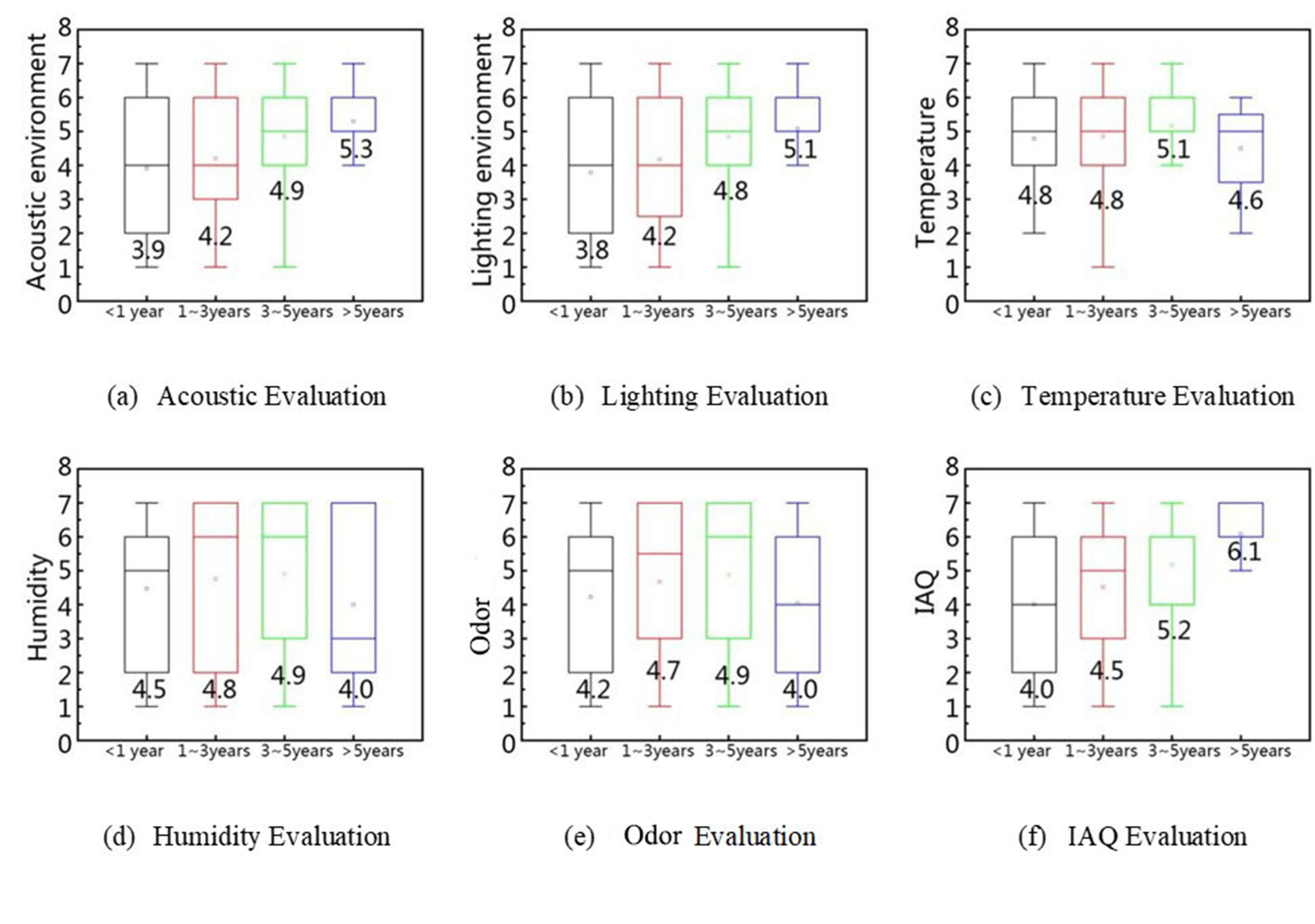

Supplement: Supplementary file 8 [file Image_7.jpg]
